# Supplementary material for: Replacing meat with alternative plant-based products (RE-MAPs): protocol for a randomised controlled trial of a behavioural intervention to reduce meat consumption
Source: BMJ Open. 2019 Jun 1;9(5):e027016. doi: 10.1136/bmjopen-2018-027016 (PMC6549643; doi:10.1136/bmjopen-2018-027016)
Supplement: Supplementary data [file bmjopen-2018-027016supp001.pdf]

NUFFIELD DEPARTMENT OF PRIMARY CARE HEALTH SCIENCES

Principal Researcher: Filippo Bianchi
Contact Details: RE-MAP@phc.ox.ac.uk
Departmental web page: www.phc.ox.ac.uk
Department's address: Nuffield Department of Primary Care health Sciences, Radcliffe Observatory Quarter, Woodstock Road, Oxford. OX2 6GG
University web page: www.ox.ac.uk

Participant ID: ___________________________________

Replacing **M**eat with **A**lternative **P**rotein **S**ources (RE-MAP STUDY)

Participant Consent Form. CUREC-Approval Reference: R54329/RE001

The purpose of this study is to test the effectiveness of a behavioural intervention designed to help people reduce their consumption of meat.

Please initial the boxes to confirm you agree

I confirm that I have read and understood the information sheet for the above study. I have had the opportunity to consider the information, ask questions and have had these answered satisfactorily.	☐
I understand that my participation is voluntary and that I am free to withdraw at any time, without having to give any reason, and without any adverse consequences.	☐
I understand that designated individuals may look at research data collected during the study where it is relevant to my taking part in this study. I give permission for these individuals to access my data.	☐
I understand that this project has been reviewed by, and received ethics clearance through, the University of Oxford Central University Research Ethics Committee.	☐
I understand who will have access to personal data provided, how the data will be stored, and what will happen to the data at the end of the project.	☐
I understand that three finger prick blood samples will be taken from me during this study to measure blood cholesterol. I understand the procedure that will be used to carry out these analyses.	☐

I understand this research will be written up as a student's thesis, I understand how personal data included in that thesis will be published and stored. ☐

I understand that I may be quoted in an anonymous way in publications pertinent to this study and that I will *not* be identified personally in any of these publications. ☐

I understand how to raise a concern or make a complaint. ☐

I consent to being audio recorded. ☐

I understand how audio recordings will be used in research outputs ☐

I consent to take part in the above study. ☐

Optional: Should I be allocated to the intervention group, I understand and consent for my name, address, telephone number, and selection of meat substitutes to be shared with Sainsbury's to carry out the food deliveries. ☐

Optional: I agree for research data collected in this study to be given to researchers, including those working outside of the EU, to be used in other research studies. I understand that any data that leave the research group will be fully anonymised so that I cannot be identified. ☐

______________________	____________	____________________________
Name of participant	Date	Signature
______________________	____________	____________________________
Name of person taking consent	Date	Signature
