# Supplementary material for: Replacing meat with alternative plant-based products (RE-MAPs): protocol for a randomised controlled trial of a behavioural intervention to reduce meat consumption
Source: BMJ Open. 2019 Jun 1;9(5):e027016. doi: 10.1136/bmjopen-2018-027016 (PMC6549643; doi:10.1136/bmjopen-2018-027016)
Supplement: Author's manuscript [file bmjopen-2018-027016.draft_revisions.pdf]

BMJ Open

BMJ Open is committed to open peer review. As part of this commitment we make the peer review history of every article we publish publicly available.

When an article is published we post the peer reviewers' comments and the authors' responses online. We also post the versions of the paper that were used during peer review. These are the versions that the peer review comments apply to.

The versions of the paper that follow are the versions that were submitted during the peer review process. They are not the versions of record or the final published versions. They should not be cited or distributed as the published version of this manuscript.

BMJ Open is an open access journal and the full, final, typeset and author-corrected version of record of the manuscript is available on our site with no access controls, subscription charges or pay-per-view fees (http://bmjopen.bmj.com).

If you have any questions on BMJ Open's open peer review process please email info.bmjopen@bmj.com

BMJ Open

Replacing meat with alternative plant-based products (RE-MAPs): Protocol for a randomized controlled trial of a behavioural intervention to reduce meat consumption

Journal:	*BMJ Open*
Manuscript ID	bmjopen-2018-027016
Article Type:	Protocol
Date Submitted by the Author:	01-Oct-2018
Complete List of Authors:	Bianchi, Filippo; University of Oxford, Nuffield Department of Primary Care Health Science<br>Aveyard, Paul; University of Oxford, Primary Care Health Sciences<br>Astbury, Nerys; University of Oxford, Nuffiled Department of Primary Care Health Sciences<br>Cook, Brian; University of Oxford, Primary Care Health Sciences<br>Cartwright, Emma; Nanyang Technological University<br>Jebb, Susan; Univerof Oxford, Primary Care Health Sciences
Keywords:	PUBLIC HEALTH, NUTRITION & DIETETICS, Planetary Health, Meat consumption, Randomised Controlled Trial, Protocol

SCHOLARONE™
Manuscripts

For peer review only

Replacing meat with alternative plant-based products (RE-MAPs): Protocol for a randomized controlled trial of a behavioural intervention to reduce meat consumption

Filippo Bianchi[1*], Paul Aveyard[1], Nerys Astbury[1], Brian Cook[1], Emma Cartwright[1], Susan A Jebb[1]

[1] Nuffield Department of Primary Care Health Science

* Corresponding Author: Filippo Bianchi, filippo.bianchi@phc.ox.ac.uk

Abstract

Introduction: Reducing meat consumption could contribute towards preventing some chronic conditions and protecting the natural environment. Meat-alternatives could help to promote a shift towards more plant-based diets, but attitudes and consumption of these foods remain low in many developed countries. This study will examine the effectiveness of a behavioural intervention to reduce meat consumption.

Methods and analyses: Re-MAP is a randomised controlled trial comparing a behavioural intervention to reduce meat consumption with a no intervention control condition. Eligible volunteers will be recruited from the general public through advertisement and randomised in a 1:1 ratio to receive no intervention or a four-week intervention comprising the provision of free plant-based meat-alternatives, written information on the health and environmental benefits of eating less meat, success stories of people who reduced their meat consumption, and recipes. The primary outcome is the change in meat consumption at four weeks (T1) from baseline. Secondary and exploratory outcomes include changes in meat consumption at eight weeks (T2) from baseline and changes from the baseline to both follow-up in other aspects of participants diet, putative psychosocial determinants of eating a low meat diet and of using meat-substitutes, and biomarkers of health risk, including blood lipid profiles, blood pressure, weight, and body composition. Linear models will be employed to explore whether the changes in each of the aforementioned outcomes differ significantly between the control and intervention group. Qualitative interviews on a subsample of participants receiving the intervention will evaluate their experiences of the intervention and help to identify the mechanisms through which the intervention reduced meat consumption or the barriers preventing the intervention to aid this dietary transition.

Ethics and dissemination: The trial has been granted ethical approval by the Medical Sciences Interdivisional Research Ethics Committee (IDREC) of the University of Oxford (Ref: R54329/RE001). All results originating from this study will be submitted for publication in scientific journals and presented to professionals and to the public at meetings and through the media.

Trial registration number: ISRCTN13180635, Pre-recruitment.

For peer review only - http://bmjopen.bmj.com/site/about/guidelines.xhtml

Strengths and limitations of this study

Strengths

- The first randomised controlled trial assessing the behavioural, nutritional, psychosocial, and health impact of a four-week intervention to reduce meat consumption through replacement with plant-based alternatives
- Assessment of putative psychosocial determinants of consumption of meat and plant-based alternatives together with qualitative interviews will help to identify the active components of the intervention and will help inform future intervention development
- Health risk outcomes will provide preliminary evidence on potential health implications of replacing meat with meat-alternatives in the diet

Limitations

- Recruitment will occur among adult-only households within Oxford (UK), limiting the generalizability of the results
- The study will not provide insights about the longer term implications of the behavioural intervention
- The study will only provide proof of principle for the effectiveness of a behavioural intervention to reduce meat consumption and future work will be needed to translate these insights into interventions in routine settings

For peer review only

For peer review only - http://bmjopen.bmj.com/site/about/guidelines.xhtml

Introduction

While meat is a source of important nutrients, red and processed meat consumption is also associated with an increased risk of developing some forms of cancer (1–3), cardiovascular disease (4–6), and type-2-diabetes (6–8). Furthermore, producing meat negatively affects the natural environment and contributes to anthropogenic global warming (9–11), which may also detrimentally affect human health on a global scale (12–15). Reducing meat consumption could therefore help to promote public health and protect the natural environment, but a recent report identified "a remarkable lack of policies, initiatives or campaigns" designed to tackle the demand for meat (16). This state of inaction is partly due to the scarcity of evidence on the effectiveness of interventions to reduce meat consumption (16–18) warranting more experimental research to develop and evaluate such interventions. The rising availability of alternatives, such as plant (including fungal) alternatives (19) could help to reduce meat consumption, as these products resemble meat in their gastronomic function, appearance, and preparation. Nevertheless, uptake of plant-based alternatives remains low in many developed countries (19–22), which might partly be due to the lack of familiarity with these foods (20,23,24). Interventions increasing people's familiarity with meat-alternatives could therefore help overcome this familiarity barrier and, in turn, help to reduce meat consumption. A recent systematic review of experimental studies concluded that interventions that supplied plant-based alternatives were associated with reductions in meat consumption during, and several weeks after the interventions (25). Nevertheless, this evidence is based on small uncontrolled pre-post intervention studies (26,27) and more systematic evaluations of the behavioural impact of such interventions is warranted. Additionally, there is currently no evidence from randomised trials on the psychosocial and health consequences of interventions aiming at reducing meat consumption through the replacement with plant-based alternatives.

Objectives

The primary aim of the Replacing Meat with Alternative Plant-based products (RE-MAP) trial is to examine the effectiveness of a behavioural intervention to reduce meat consumption compared to a no intervention control condition. Additionally this study will evaluate the impact of the same intervention on the consumption of other food groups, the nutritional composition of participants' diets, the putative psychosocial determinants of eating a low meat diet and of using plant-based meat-alternatives, and on biological markers of health risk, including blood lipid profiles, blood pressure, weight, and body composition. This study also aims to qualitatively investigate participants' experiences of the intervention and the mechanisms through which the intervention reduced meat consumption or the barriers preventing the intervention to aid this dietary transition.

For peer review only - http://bmjopen.bmj.com/site/about/guidelines.xhtml

Methods

Study design and setting

The Re-MAP study will employ a two-arm parallel group individually randomised controlled trial to evaluate a four-week behavioural intervention to reduce meat consumption. The primary endpoint is defined as the change in average daily meat consumption at four weeks form baseline, assessed through self-reported seven days food diaries. The study will be conducted in Oxford, United Kingdom.

Recruitment

Participants will be recruited from the general population through advertisements in public buildings, newspapers, mailing lists, and social media. Individuals contacting the study team will receive a written information sheet summarizing the study protocol. Individuals confirming their interest will be called by the recruiting member of the research team, who will summarise the study protocol and answer any outstanding question. The recruiting member of the research team will also screen individuals against the eligibility criteria and invite eligible individuals to attend an enrolment appointment.

Eligibility criteria

Inclusion criteria:

(a) are ≥18 years old
(b) self-report to eat meat regularly
(c) belong to an adult-only household
(d) are willing to try meat-alternatives
(e) own adequate food storing facilities
(f) possess a device compatible with the requirements of the online food diary
(g) provide informed consent

Exclusion criteria:

(a) report they have relevant food allergies
(b) report suffering from an eating disorder
(c) report to be pregnant or plan pregnancy in the study period
(d) belong to the same household as a previously enrolled participant
(e) report consuming meat-alternatives more than once a week on average
(f) return baseline dietary records of insufficient quality for analysis
(g) the recruiting researcher deems the interested individual unable to adhere appropriately to the study protocol (e.g. insufficient knowledge of the English language, planned absences from main residence during the course of the study, enrolled in other longitudinal dietary intervention study).

Participant flow

Enrolment appointment

For peer review only - http://bmjopen.bmj.com/site/about/guidelines.xhtml

The enrolment appointment will take place on University premises. During this appointment an appropriately trained member of the research team will seek written informed consent, witnessing this by means of dated signature. After gaining informed consent the enrolling member of the research team will set up participants' online food diaries to include six possible meal entries per day (breakfast, mid-morning, lunch, mid-afternoon, dinner, and post-dinner) and to allow the research team to remotely access participants' food diaries by means of a password.

Baseline

Following the enrolment appointment, participants will complete a seven-day food diary over the week leading up to the following appointment, the baseline (T0). Participants not keeping sufficiently detailed diaries and those eating meat on less than five eating occasions over the week will be discontinued. At the baseline appointment an appropriately trained member of the research team will collect participants' food diaries, ask participants to answer the baseline online questionnaire, and measure participants blood lipids profile, blood pressure, weight, and body composition. At the end of the baseline appointment participants will be randomised to one of the two study conditions and will then follow the respective protocol for the next four weeks.

Follow up

Participants will be invited to attend a four-week (T1) and an eight-week (T2) follow-up and to keep a seven-days food diary over the week leading up to each follow-up. During the follow-up appointments a member of the research team will collect the respective food diary, ask participants to answer an online questionnaire, and measure participants blood lipids profile, blood pressure, weight, and body composition.

Sample size

Due to lack of research studies directly comparable to ours, pragmatic considerations have guided the decision to terminate recruitment once a sample of at least 100 volunteers will have completed the four-weeks follow-up. A power analysis based on this pragmatically selected sample size suggests that 100 participants completing the primary outcome will allow detecting a medium effect size of d=0.6 with a power of 1-beta=0.84 and a two-tailed alpha criterion of 0.05.

Randomisation and blinding

Participants' group allocation will be based on a computer generated randomisation sequence, produced by an independent statistician. The randomisation sequence was designed to individually allocate participants to the intervention or control condition in a 1:1 ratio and to achieve a proportional gender balance in the two conditions through blocking and stratification by sex. The research team is blinded to the randomisation sequence and to its block sizes and sequence. Allocation will be revealed to the researcher performing the randomisation only after the first food diary has been returned. Due to the nature of the intervention, participants and some

For peer review only - http://bmjopen.bmj.com/site/about/guidelines.xhtml

members of the research team cannot be blind to participants' group allocation. Other members of the research team analysing the food diaries will be blind to the group allocation.

Intervention and comparator

Intervention

Re-MAP (replacing meat with alternative plant-based products) is a four-week behavioural intervention, which aims to reduce meat consumption, defined as non-seafood meat products, among regular meat eaters. Following an analysis of the target behaviour, i.e. a reduction in meat consumption, we included five psychosocial variables as the intervention's targets: attitudes, perceived behavioural control, and subjective social norms of eating a low meat diet, as well as attachment to meat, and eating identities. We then selected four intervention functions from the Behaviour Change Wheel (28,29) with the aim of influencing these psychosocial variables: (1) environmental restructuring enacted through providing meat-alternatives for four weeks, (2) training enacted through recipes, (3) education enacted through infographics on the health and environmental benefits of eating less meat, and (4) social modelling enacted through written vignettes outlining the story of people who reduced their meat consumption. These success stories were developed following an online patient and public involvement (PPI) activity. This PPI activity involved asking people who consciously reduced their consumption of meat to share their motives to do so, their strategies to enact this dietary transition, and the way they overcame the challenges associated with this transition. A logic model of the intervention is displayed in figure 1.

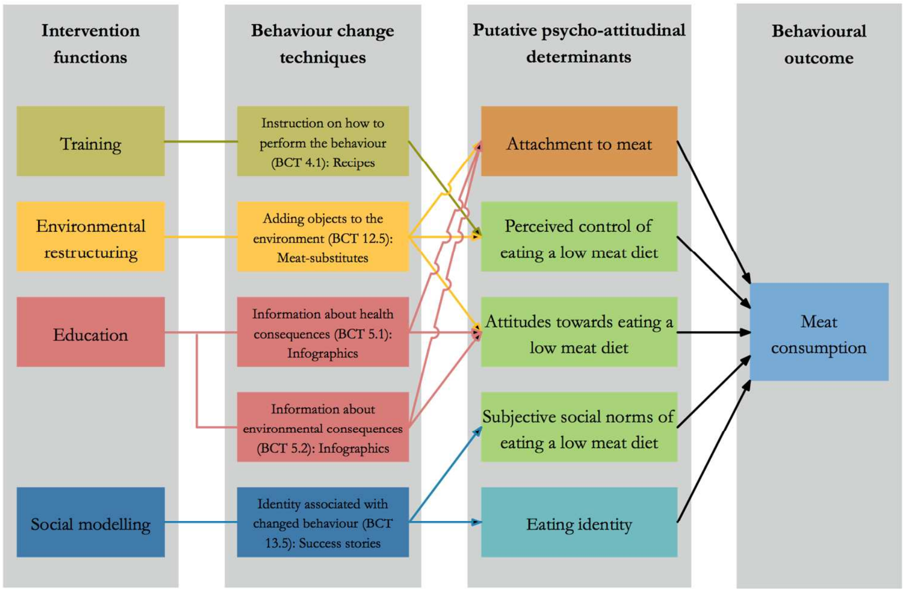

Figure 1: Intervention logic model

Following the development of the basic intervention structure, we held a discussion group with ten members of the general public aiming to improve the acceptability and effectiveness of the RE-MAP intervention. We invited five meat eaters and five

For peer review only - http://bmjopen.bmj.com/site/about/guidelines.xhtml

meat reducers to attend the discussion group, aiming to include people representing the target population of the intervention as well as people that successfully reduced their meat consumption. Public contributors were recruited using an established mailing list. The discussion group informed the development of each intervention component including:

- What type of meat-alternatives to offer as part of the intervention
- How to make the infographics about the health and environmental benefits of eating less meat more engaging and easily accessible to different publics
- What language to use as part of our success stories vignettes and how to increase their relatability
- What cookbooks and recipes to use as part of our intervention

Comparator

Participants in the control condition will receive no intervention. The TiDIER checklist (30) for the Re-MAP intervention and the comparator is reported in table 1.

	Intervention	Comparator
BRIEF NAME	Re-MAP – a behavioural intervention to reduce meat consumption	No intervention
WHY	Environmental restructuring: Meat alternatives were provided for one month with the aim of enhancing attitudes towards and behavioural control of eating a low meat diet by making meat-free alternative easily available. This intervention component also aimed to reduce participants' attachment to meat.<br><br>Training: Recipes were provided with the aim of enhancing participants' behavioural control of eating a low meat diet by enhancing their skills of preparing meat-free meals<br><br>Education: Information leaflets about the health and environmental benefits of eating less meat were provided to enhance participants' attitudes towards eating a low meat diet and to reduce participants' attachment to meat.<br><br>Social modelling: written success stories of people who reduced their meat consumption were provided to increase participants perceived social norm of eating a low meat diet and to promote the dietary identity of meat reducers, such as flexitarians.	N/A
WHAT	Environmental restructuring: Participants were provided with meat alternatives for one month. Participants were provided with a printed catalogue of commercially available meat-alternatives and were asked to select enough meat-alternatives to have a meat-free products available on every occasion on which they would normally have meat. Participants were free to order enough foods to cater for themselves and other members in their household, if they wished to do so. The meat substitutes were delivered to participants' homes by a food retailer on two occasions over the intervention month: the first delivery was scheduled immediately after participants were allocated to the intervention condition, while the second delivery was scheduled two weeks after.<br><br>Training: A printed booklet containing 11 illustrated recipes of meat-alternatives was delivered immediately after participants were allocated to the intervention condition. A second cookbook predominantly reporting on more general meat-free recipes (i.e. not focussing on meat alternatives) was	N/A

For peer review only - http://bmjopen.bmj.com/site/about/guidelines.xhtml

	provided during the fourth intervention week.<br><br>Education: Participants received 10 printed pages of illustrated information on the health and environmental benefits of eating less meat were delivered. Immediately after being allocated to the to the intervention condition participants received an illustrated binder in which to collect the information leaflets, including the first 2 pages of introductory information and the academic sources from which the information was drawn. Participants were then send the leaflets on the health benefits (4 pages) and environmental benefits (4 pages) of eating less meat delivered per post to their home over the course of the intervention month.<br><br>Success stories: Participants received three illustrated success stories vignettes, which were delivered per post to their home during the last intervention week. Participants also received a sheet on which they could report their own success story if the wish to do so. Participants were asked to add this information to their illustrated binder.	
WHO	The lead researcher of this trial (FB) delivered the intervention. An Access Database System was used to schedule the deliveries of each intervention component ensuring that each intervention component was delivered at the appropriate time for each participant.	N/A
HOW	The intervention consistent in the delivery of the aforementioned materials. We used the delivery services of one of UK's largest food retailers to purchase and deliver the meat-alternatives to participants. We used Royal Mail to send the printed material. The binder was delivered to participants immediately they were randomised to the intervention condition.	N/A
WHERE	N/A	N/A
TAILORED	N/A	N/A
HOW Well	We elected to use a single study account with the food retailer to schedule all the study deliveries, which enabled us to monitor the successful completion and receipt of each delivery. Due to the nature of the intervention we did not consider it necessary to establish any other systems to monitor the fidelity of the intervention delivery.	N/A

For peer review only

Outcomes

Primary outcome

- Change in mean daily grams of meat consumed between the baseline (T0) and the four-week follow-up (T1)

Secondary outcomes

- Change in mean daily grams of meat consumed between the baseline (T0) and the eight-week follow-up (T2)
- Change in intention to eat a low meat diet between the baseline (T0) and both follow-up (T1, T2)
- Change in attachment to meat, eating identities, and in attitudes, perceived behavioural control, and subjective social norm of eating a low meat diet between the baseline (T0) and both follow-up (T1, T2)

Exploratory outcomes

- Change in participants' blood lipid profiles (total cholesterol, HDL cholesterol, triglycerides, LDL cholesterol, non-HDL cholesterol, LDL:HDL cholesterol ratio) between the baseline (T0) and both follow up (T1, T2)

For peer review only - http://bmjopen.bmj.com/site/about/guidelines.xhtml

- Change in systolic and diastolic blood pressure between the baseline (T0) and both follow up (T1, T2)
- Change in participants' body mass index between the baseline (T0) and both follow up (T1, T2)
- Change in participants' body fat percentage baseline (T0) and both follow up (T1, T2)
- Change in the number of meals containing foods from other food groups between the baseline (T0) and both follow up (T1, T2)
- Change in participants' mean daily energy, macro-, and micronutrients intake between the baseline (T0) and both follow up (T1, T2)
- Change in participants' intentions, attitudes, perceived behavioural control, and subjective social norms of using meat-alternatives between the baseline (T0) and both follow up (T1, T2)
- Change in participants' desire for meat-substitutes to be similar to meat between the baseline (T0) and both follow up (T1, T2)

Measurements

Table 2 provides a summary of the measurement collected.

	Visits				
	Telephone screening	Enrolment Visit	Baseline Visit	4 week follow up	8 week follow up
Enrolment					
Eligibility screening	X				
Informed consent		X			
Randomisation			X		
Intervention					
REMAP					
Control					
Demographic and psychosocial traits					
Demographics			X		
Food neophobia			X		
Self control scale			X		
Dietary measurements					
Food diary			X	X	X
Retrospective eating questionnaire			X	X	X
Psychosocial variables					
Attitude towards eating a low meat diet and using meat-alternatives			X	X	X
Perceived behavioural control of eating a low meat diet and using meat-alternatives			X	X	X
Subjective social norm of eating a low meat diet and using meat-alternatives			X	X	X
Intention to eat a low meat diet and to use meat-alternatives			X	X	X
Attachment to meat			X	X	X
Eating identity			X	X	X
Desire for similarity of meat and alternatives			X	X	X
Biophysical outcomes					

For peer review only - http://bmjopen.bmj.com/site/about/guidelines.xhtml

Height			X		
Weight			X	X	X
Body composition			X	X	X
Blood pressure			X	X	X
Blood lipids profile			X	X	X
Qualitative work-stream					
Semi structured interviews					X

Socio-demographic characteristics

At the baseline only, participants will be asked to self-report on their age, sex, highest degree, household income, household composition, ethnicity, and nationality.

Psychological trait characteristics

- At the baseline only, participants' trait food neophobia will be measured using questionnaire scale adapted from Pliner and Hobden (31) including six items with a 7-point scale (disagree strongly – agree strongly).
- At the baseline only, participants' self-control will also be assessed using a questionnaire scale adapted from Tangney et al. (32) including eight items with a 7-point scale (disagree strongly – agree strongly).

Dietary measurements

- Meat consumption will be measured in grams/day by disaggregating meat products recorded by participants on their seven-days food diaries. The daily average will exclude days in which energy intake was <1000kcal which are considered unlikely to represent habitual consumption
- Average daily number of meals containing foods from other food groups will be measured counting the meals in participants' food diaries containing the food groups of interest, including:
    - Unprocessed pork meat
    - Unprocessed red meat from ruminants
    - Unprocessed poultry or game meat
    - Processed meat
    - Mycoprotein meat-alternatives
    - Soy-based meat alternatives or meat alternatives made of other textured vegetable protein
    - Other meat-alternatives
    - Milk and yoghurt
    - Cheese
    - Dairy-free milk- and yoghurt-alternatives
    - Dairy-free cheese-alternatives
    - Fish and seafood
    - Eggs
    - Pulses
    - Vegetables other than those in meat-alternatives
    - Starchy foods other than those in meat-alternatives
    - Nuts and seeds other than those in meat-alternatives

For peer review only - http://bmjopen.bmj.com/site/about/guidelines.xhtml

- Fruit
- Savoury and sweet snacks
- Soft drinks
- Alcoholic drinks

The same outcome will be measured with a retrospective eating questionnaire, asking participants to recall the number of eating occasions on which they had the foods listed above over the same week of the food diary. The latter measure will be used in sensitivity analysis.

- The daily average energy intake and nutritional composition of participants' diets will be measured using data from the online food diary.

Psychosocial variables

- Attachment to meat will be measured using the meat attachment questionnaire (33).
- Eating identities will be self-reported by participants among meat-eater, omnivore, flexitarian, pescatarian, vegetarian, vegan, or 'other'. The identities that involve no consumption of non-seafood meat (i.e. pescatarian, vegetarian, and vegan) will be clustered together in non-meat eating dietary identities.
- Attitudes, subjective social norms, and perceived behavioural control to eat a low meat diet and to use meat-alternatives will be respectively assessed with three questionnaire items constructed following Francis et al. (34) on a 7-point scale (disagree strongly – agree strongly).
- Intention to eat a low meat diet and to use meat substitutes will be assessed using a single questionnaire item on a on a 7-point scale (disagree strongly – agree strongly).
- Desire for similarity between meat and meat substitutes will be assessed using 11 questionnaire items with a 7-point scale (disagree strongly – agree strongly) adapted from Hoek et al. (20)

Physical measures

- Blood lipids profiles (total cholesterol, HDL cholesterol, triglycerides, LDL cholesterol, non-HDL cholesterol, LDL:HDL cholesterol ratio) will be measured using Alere Cholestech LDX®
- Height will be measured to the nearest 0.1cm using a stadiometer
- Weight and body composition will be measured using an electronic scale (SC-240 MA, Tanita Japan), which records the proportion of body fat using bioelectrical impedance. Weight was recorded to the nearest 0.1 kg
- Seated blood pressure will be measured as the average of the second and third reading of three seated readings

Retention

We will use reminder text messages to increase attendance to each of the four study appointments. Additionally participants will receive financial compensation for partaking in each of the 3 assessment visits. Participants will have the right to

For peer review only - http://bmjopen.bmj.com/site/about/guidelines.xhtml

withdraw from the study at any time. The principal investigator will have the right to discontinue participants' involvement in the study when they become ineligible and/or when significant protocol deviations occur. The data of withdrawn participants will be kept and used in exploratory and sensitivity analyses, unless specifically requested otherwise.

Adverse events

Any study-related adverse event will be reported to the Research Ethics Committee in accordance to Good Clinical Practice (GCP). All study-related adverse events will be included in the final trial report.

Data management

Data will be entered by a trained member of the research team and stored in an OpenClinica database that was specifically developed for this trial and features ranges and validation checks to promote reliability in the data entry process. Data recording and storage will run in accordance with GCP.

Statistical analyses

We will employ linear models to investigate whether changes in meat consumption between the baseline and both follow up differ significantly between the intervention and the control group. Our main analysis will employ unadjusted models and only include data from participants completing the relevant follow-up. Sensitivity analysis will be performed with a Baseline Observation Carried Forward (BOCF) assumption for missing data and adjusting for baseline variables. The intervention effect will be reported with 95% CI and p-values. A two-tailed criterion p-value of alpha=0.05 will be used to assess the statistical significance of the results. The same procedure will be employed to assess whether changes in the other pre-specified dietary, nutritional, psychosocial, and biophysical outcomes between the baseline and both follow-up differ significantly between the control and the intervention group. Detailed main-, subgroup-, and sensitivity analyses plans will be finalised before conducting any specific outcome analysis. No interim analysis is planned.

Qualitative study

After the eight-week follow-up, a subsample of participants receiving the intervention will be invited to take part in a semi-structured interview. This qualitative study is aimed at understanding participants' experiences of the intervention and the mechanisms through which the intervention helped them reduce their meat consumption or the barriers that prevented the intervention to aid this transition. The semi-structured interviews will follow a discussion guide while also remaining sensitive to unsolicited themes. The interview will set the context by asking participants to elaborate on their motivation to volunteer for the trial and on their thoughts and feelings towards reducing meat consumption prior to enrolling into the study. Participants will then be encouraged to elaborate on the mechanisms through which they felt that the intervention helped them eat less meat or the barriers that prevented the intervention to do so. In doing so participants will be

For peer review only - http://bmjopen.bmj.com/site/about/guidelines.xhtml

prompted to think about the intervention in its entirety as well as about each individual intervention component. Participants will also be encouraged to elaborate on their perceived ability and motivation to maintain a lower consumption of meat after the intervention period and beyond the context of the study. Whenever possible we will use open questions to encourage participants to elaborate on their thoughts and feelings freely and in depth. We aim to avoid questions of evaluative nature to minimise the risk of social desirability bias. We anticipate interviewing 20 participants, however sampling will be extended should new themes emerge during the interviewing process. We will employ a purposeful sampling technique to achieve a sex balance. Participants will be free to decide whether to be interviewed and no additional compensation will be offered to participants agreeing to be interviewed. Qualitative interviews will be conducted in person and transcribed verbatim. Transcriptions will be analysed using NVIVO and employing a data driven thematic analysis to identify codes and to group these codes into broader themes.

Trial steering committee

The principal investigator will be responsible for the project coordination and the senior investigators will oversee the operational aspects of the trial. The authors of this protocol will form the trial management group (TMG), which will regularly monitor the study implementation, as well as the data generation, documentation, and reporting. All members of the TMG are trained in GCP and will take appropriate actions to safeguard participants and the quality of the trial. Access to data will be granted to appropriate members of the research team and to authorised representatives from the host institution to monitor and/or audit the study and ensure compliance with regulations.

Ethics and dissemination

The investigators will ensure that this study is conducted in accordance with the principles of the Declaration of Helsinki, with relevant institutional regulations, with GCP and GDPR regulations. This study was reviewed and received ethical approval (R54329/RE001) by the Medical Sciences Interdivisional Research Ethics Committee of the University of Oxford. Substantial planned changes to the protocol, an end of study notification, and a final report will be submitted to the aforementioned research ethics committee. The results of this RCT will be reported following the Consolidated Standards of Reporting Trials guidelines (35) and submitted for publication to scientific journals, regardless of the research outcome. Authorship will be determined in accordance with the ICMJE guidelines. Contributors of other parties and funding will be acknowledged. Results will also be presented at national and international conferences and disseminated through established networks. A lay summary will be distributed through an established newsletter to which participants can subscribe on their last study appointment.

Acknowledgements

We thank all the PPI contributors for having helped us develop the RE-MAP intervention. We thank Lynne Maddocks for her assistance in forming the PPI panel

For peer review only - http://bmjopen.bmj.com/site/about/guidelines.xhtml

for this study. We thank Lucy Eldridge for her support in developing the study database. We thank Jason Oke for his assistance in developing the randomization sequence.

Authors' contributions: All authors have been involved in shaping each stage of this research protocol. FB, SAJ, and PA have designed the study. FB has written this protocol and developed the intervention. FB and NA have developed the trial management system. NA, BC, and EC have contributed in designing this research and the intervention.

Funding: This research is funded by the Wellcome Trust, Our Planet Our Health programme (Livestock, Environment and People - LEAP), award number 205212/Z/16/Z. FB's time on this project is funded by the Medical Research Council (MRC), Green Templeton College Oxford, and the National Institute for Health Research (NIHR) School for Primary Care Research (SPCR). EC's and BC's time on this project is funded by the Wellcome Trust, Our Planet Our Health programme (Livestock, Environment and People - LEAP), award number 205212/Z/16/Z. NA, PA, and SAJ are supported by the NIHR Oxford Biomedical Research Centre and Collaboration for Leadership in Applied Health Research and Care Oxford at Oxford Health NHS Foundation Trust. PA and SAJ are NIHR Senior Investigators.

Competing interests statement

The authors have no known competing interests to declare.

References

1. Bouvard V, Loomis D, Guyton KZ, Grosse Y, Ghissassi F El, Benbrahim-Tallaa L, et al. Carcinogenicity of consumption of red and processed meat. Lancet Oncol [Internet]. Elsevier; 2015 Dec 1 [cited 2017 Nov 9];16(16):1599–600. Available from: http://linkinghub.elsevier.com/retrieve/pii/S1470204515004441
2. Chan DSM, Lau R, Aune D, Vieira R, Greenwood DC, Kampman E, et al. Red and processed meat and colorectal cancer incidence: meta-analysis of prospective studies. PLoS One [Internet]. Public Library of Science; 2011 Jan 6 [cited 2015 Oct 26];6(6):e20456. Available from: http://journals.plos.org/plosone/article?id=10.1371/journal.pone.0020456
3. Parkin DM, Boyd L, Walker LC. 16. The fraction of cancer attributable to lifestyle and environmental factors in the UK in 2010. Br J Cancer [Internet]. 2011 Dec 6 [cited 2014 Oct 30];105 Suppl:S77-81. Available from: http://www.pubmedcentral.nih.gov/articlerender.fcgi?artid=3252065&tool=

For peer review only - http://bmjopen.bmj.com/site/about/guidelines.xhtml

pmcentrez&rendertype=abstract

4. Bechthold A, Boeing H, Schwedhelm C, Hoffmann G, Knüppel S, Iqbal K, et al. Food groups and risk of coronary heart disease, stroke and heart failure: A systematic review and dose-response meta-analysis of prospective studies. Crit Rev Food Sci Nutr [Internet]. 2017 Oct 17 [cited 2017 Nov 9];1–20. Available from: http://www.ncbi.nlm.nih.gov/pubmed/29039970

5. Micha R, Wallace SK, Mozaffarian D. Red and Processed Meat Consumption and Risk of Incident Coronary Heart Disease, Stroke, and Diabetes Mellitus: A Systematic Review and Meta-Analysis. Circulation [Internet]. 2010 Jun 1 [cited 2017 May 23];121(21):2271–83. Available from: http://www.ncbi.nlm.nih.gov/pubmed/20479151

6. Micha R, Michas G, Mozaffarian D. Unprocessed red and processed meats and risk of coronary artery disease and type 2 diabetes--an updated review of the evidence. Curr Atheroscler Rep [Internet]. 2012 Dec [cited 2015 Dec 27];14(6):515–24. Available from: http://www.pubmedcentral.nih.gov/articlerender.fcgi?artid=3483430&tool=pmcentrez&rendertype=abstract

7. Feskens EJM, Sluik D, van Woudenbergh GJ. Meat Consumption, Diabetes, and Its Complications. Curr Diab Rep [Internet]. 2013 Apr 25 [cited 2017 Nov 9];13(2):298–306. Available from: http://www.ncbi.nlm.nih.gov/pubmed/23354681

8. Barnard N, Levin S, Trapp C. Meat consumption as a risk factor for type 2 diabetes. Nutrients [Internet]. Multidisciplinary Digital Publishing Institute (MDPI); 2014 Feb 21 [cited 2017 Nov 9];6(2):897–910. Available from: http://www.ncbi.nlm.nih.gov/pubmed/24566443

9. Steinfeld H, Gerber P, Wassenaar T. Livestock's long shadow. Issues Options, … [Internet]. 2006 [cited 2017 May 23]; Available from: https://www.rfp-europe.org/fileadmin/SITE_ERFP/ERFP_meetings/2007_Dublin/Dublin2007_LivestocksLongShadow_HOFFMANN.pdf

10. Pimentel D, Pimentel M. Sustainability of meat-based and plant-based diets and the environment. Am J Clin Nutr [Internet]. American Society for Nutrition; 2003 Sep [cited 2017 May 23];78(3 Suppl):660S–663S. Available from: http://www.ncbi.nlm.nih.gov/pubmed/12936963

11. Tilman D, Clark M. Global diets link environmental sustainability and human health. Nature [Internet]. Nature Publishing Group, a division of Macmillan

For peer review only - http://bmjopen.bmj.com/site/about/guidelines.xhtml

Publishers Limited. All Rights Reserved.; 2014 Nov 27 [cited 2015 Jul 27];515(7528):518–22. Available from: http://dx.doi.org/10.1038/nature13959

12. Watts N, Amann M, Ayeb-Karlsson S, Belesova K, Bouley T, Boykoff M, et al. The Lancet Countdown on health and climate change: from 25 years of inaction to a global transformation for public health. Lancet [Internet]. Elsevier; 2017 Oct 30 [cited 2018 Jan 25]; Available from: https://www.sciencedirect.com/science/article/pii/S0140673617324649?_rdoc=1&_fmt=high&_origin=gateway&_docanchor=&md5=b8429449ccfc9c30159a5f9aeaa92ffb

13. Mcmichael AJ, Campbell-Lendrum DH, Corvalán CF, Ebi KL, Githeko AK, Scheraga JD, et al. Climate change and human health. Risks and responses [Internet]. 2003 [cited 2018 Jan 25]. Available from: http://www.who.int/globalchange/publications/climchange.pdf

14. Economou V, Gousia P. Agriculture and food animals as a source of antimicrobial-resistant bacteria. Infect Drug Resist [Internet]. Dove Press; 2015 [cited 2018 Jan 25];8:49–61. Available from: http://www.ncbi.nlm.nih.gov/pubmed/25878509

15. Tang KL, Caffrey NP, Nóbrega DB, Cork SC, Ronksley PE, Barkema HW, et al. Restricting the use of antibiotics in food-producing animals and its associations with antibiotic resistance in food-producing animals and human beings: a systematic review and meta-analysis. Lancet Planet Heal [Internet]. Elsevier; 2017 Nov 1 [cited 2018 May 13];1(8):e316–27. Available from: http://www.ncbi.nlm.nih.gov/pubmed/29387833

16. Bailey R, Froggatt A WL. Livestock–climate change's forgotten sector. [Internet]. 2014 [cited 2017 Nov 10]. Available from: https://scholar.google.co.uk/scholar?hl=en&as_sdt=0%2C5&q=Livestock+–
+Climate+Change's+Forgotten+Sector+Global+Public+Opinion+on+Meat+and+Dairy+Consumption&btnG=

17. Garnett T, Mathewson S, Angelides P, Borthwick F. Policies and actions to shift eating patterns: What works? [Internet]. 2015 [cited 2017 May 24]. Available from: http://www.fcrn.org.uk/sites/default/files/fcrn_chatham_house_0.pdf

18. Wellesley L, Happer C, Froggatt A. Chatham House Report Changing

For peer review only - http://bmjopen.bmj.com/site/about/guidelines.xhtml

Climate, Changing Diets Pathways to Lower Meat Consumption. 2015 [cited 2017 Nov 10]; Available from: https://www.chathamhouse.org/sites/files/chathamhouse/publications/research/CHHJ3820 Diet and climate change 18.11.15_WEB_NEW.pdf

19. Mintel Group Ltd. Meat-free Foods - UK [Internet]. 2017 [cited 2017 Nov 12]. Available from: http://academic.mintel.com/sinatra/oxygen_academic/list/id=796253&type=RCItem#0_1___page_RCItem=0

20. Hoek A, Luning P, Weijzen P, Engels W, Kok F. Replacement of meat by meat substitutes. A survey on person-and product-related factors in consumer acceptance. Appetite [Internet]. 2011 [cited 2017 May 24]; Available from: http://www.sciencedirect.com/science/article/pii/S0195666311000523

21. Hartmann C, Siegrist M. Consumer perception and behaviour regarding sustainable protein consumption: A systematic review. Trends Food Sci Technol [Internet]. 2017 Mar [cited 2017 Nov 26];61(61):11–25. Available from: http://linkinghub.elsevier.com/retrieve/pii/S0924224416302904

22. Kumar P, Chatli MK, Mehta N, Singh P, Malav OP, Verma AK. Critical Reviews in Food Science and Nutrition Meat analogues: Health promising sustainable meat substitutes Meat analogues: Health promising sustainable meat substitutes. 2017 [cited 2018 May 16]; Available from: http://www.tandfonline.com/action/journalInformation?journalCode=bfsn20

23. Clery E, Bailey R. Food technologies Findings from the 2008 British Social Attitudes survey. Food Technol Find from 2008 Br [Internet]. 2010 [cited 2017 May 24]; Available from: http://www.fcrn.org.uk/sites/default/files/BSA_Food_technologies_findings.pdf

24. Hoek AC, Elzerman JE, Hageman R, Kok FJ, Luning PA, Graaf C de. Are meat substitutes liked better over time? A repeated in-home use test with meat substitutes or meat in meals. Food Qual Prefer [Internet]. Elsevier; 2013 Apr 1 [cited 2018 May 13];28(1):253–63. Available from: https://www.sciencedirect.com/science/article/abs/pii/S0950329312001280

25. Bianchi F, Garnett E, Dorsel C, Aveyard P, Jebb SA. Restructuring physical micro-environments to reduce the demand for meat: A systematic review with qualitative comparative analysis. Submitted.

For peer review only - http://bmjopen.bmj.com/site/about/guidelines.xhtml

26. Clark M. Chronic Effects Of Replacing Red And Processed Meat With Non/Reduced Meat Alternatives [Internet]. Cambridge University Press; 2017 [cited 2017 Nov 12]. Available from: https://www.cambridge.org/core/product/identifier/S0029665117001434/type/journal_article

27. Holloway T, Salter AM, McCullough FS. Dietary intervention to reduce meat intake by 50% in University students – a pilot study. Proc Nutr Soc [Internet]. Cambridge University Press; 2012 Jan 19 [cited 2017 Nov 12];71(OCE2):E164. Available from: http://www.journals.cambridge.org/abstract_S0029665112002212

28. Michie S, Atkins L, West R. The behaviour change wheel: a guide to designing interventions. Needed: physician leaders [Internet]. 2014 [cited 2017 May 24]; Available from: http://www.physicianleaders.ca/assets/cspljournalsummer2015.pdf#page=26

29. Michie S, Stralen M van. The behaviour change wheel: a new method for characterising and designing behaviour change interventions. 2011 [cited 2017 May 24]; Available from: https://implementationscience.biomedcentral.com/articles/10.1186/1748-5908-6-42

30. Hoffmann TC, Glasziou PP, Boutron I, Milne R, Perera R, Moher D, et al. Better reporting of interventions: template for intervention description and replication (TIDieR) checklist and guide. BMJ [Internet]. 2014 [cited 2017 Sep 1];348. Available from: http://www.bmj.com/content/348/bmj.g1687

31. Pliner P, Hobden K. Development of a scale to measure the trait of food neophobia in humans. Appetite [Internet]. 1992 Oct [cited 2018 May 13];19(2):105–20. Available from: http://www.ncbi.nlm.nih.gov/pubmed/1489209

32. Tangney J, … AB-S-R and, 2018 undefined. High self-control predicts good adjustment, less pathology, better grades, and interpersonal success. taylorfrancis.com [Internet]. [cited 2018 May 13]; Available from: https://www.taylorfrancis.com/books/e/9781351707756/chapters/10.4324%2F9781315175775-12

33. Graça J, Calheiros MM, Oliveira A. Attached to meat? (Un)Willingness and intentions to adopt a more plant-based diet. Appetite [Internet]. 2015 Dec

For peer review only - http://bmjopen.bmj.com/site/about/guidelines.xhtml

[cited 2017 Nov 13];95:113–25. Available from: http://www.ncbi.nlm.nih.gov/pubmed/26148456

34. Francis J, Eccles M, Johnston M, Walker A. Constructing questionnaires based on the theory of planned behaviour: A manual for health services researchers. 2004 [cited 2018 May 13]; Available from: http://openaccess.city.ac.uk/1735/1/TPB Manual FINAL May2004.pdf

35. Schulz KF, Altman DG, Moher D. CONSORT 2010 Statement: updated guidelines for reporting parallel group randomised trials. BMC Med [Internet]. 2010 Jan [cited 2015 Mar 14];8(1):18. Available from: http://www.biomedcentral.com/1741-7015/8/18

For peer review only

For peer review only - http://bmjopen.bmj.com/site/about/guidelines.xhtml

BMJ Open

Replacing meat with alternative plant-based products (RE-MAPs): Protocol for a randomized controlled trial of a behavioural intervention to reduce meat consumption

Journal:	*BMJ Open*
Manuscript ID	bmjopen-2018-027016.R1
Article Type:	Protocol
Date Submitted by the Author:	15-Jan-2019
Complete List of Authors:	Bianchi, Filippo; University of Oxford, Nuffield Department of Primary Care Health Science<br>Aveyard, Paul; University of Oxford, Primary Care Health Sciences<br>Astbury, Nerys; University of Oxford, Nuffiled Department of Primary Care Health Sciences<br>Cook, Brian; University of Oxford, Primary Care Health Sciences<br>Cartwright, Emma; Nanyang Technological University<br>Jebb, Susan; Univerof Oxford, Primary Care Health Sciences
**Primary Subject Heading**:	Public health
Secondary Subject Heading:	Nutrition and metabolism
Keywords:	PUBLIC HEALTH, NUTRITION & DIETETICS, Planetary Health, Meat consumption, Randomised Controlled Trial, Protocol

SCHOLARONE™
Manuscripts

Replacing meat with alternative plant-based products (RE-MAPs): Protocol for a randomized controlled trial of a behavioural intervention to reduce meat consumption

Filippo Bianchi[1*], Paul Aveyard[1], Nerys Astbury[1], Brian Cook[1], Emma Cartwright[1], Susan A Jebb[1]

[1] Nuffield Department of Primary Care Health Science
* Corresponding Author: Filippo Bianchi, filippo.bianchi@phc.ox.ac.uk

Abstract

Introduction: Reducing meat consumption could contribute towards preventing some chronic conditions and protecting the natural environment. This study will examine the effectiveness of a behavioural intervention to reduce meat consumption.

Methods and analyses: Re-MAP is a randomised controlled trial comparing a behavioural intervention to reduce meat consumption with a no intervention control condition. Eligible volunteers will be recruited from the general public through advertisement and randomised in a 1:1 ratio to receive no intervention or a four-week intervention comprising the provision of free plant-based meat-alternatives, written information on the health and environmental benefits of eating less meat, success stories of people who reduced their meat consumption, and recipes. The primary outcome is the change in meat consumption at four weeks (T1) from baseline. Secondary and exploratory outcomes include changes in meat consumption at eight weeks (T2) from baseline and changes from the baseline to both follow-up in other aspects of participants diet, putative psychosocial determinants of eating a low meat diet and of using meat-substitutes, and biomarkers of health risk, including blood lipid profiles, blood pressure, weight, and body composition. Linear models will be employed to explore whether the changes in each of the aforementioned outcomes differ significantly between the control and intervention group. Qualitative interviews on a subsample of participants receiving the intervention will evaluate their experiences of the intervention and help to identify the mechanisms through which the intervention reduced meat consumption or the barriers preventing the intervention to aid this dietary transition.

Ethics and dissemination: The trial has been granted ethical approval by the Medical Sciences Interdivisional Research Ethics Committee (IDREC) of the University of Oxford (Ref: R54329/RE001). All results originating from this study will be submitted for publication in scientific journals and presented at meetings and through the media.

Trial registration number: ISRCTN13180635, Pre-recruitment.

For peer review only

For peer review only - http://bmjopen.bmj.com/site/about/guidelines.xhtml

Strengths and limitations of this study

Strengths

- The first randomised controlled trial assessing the behavioural, nutritional, psychosocial, and health impact of a four-week intervention to reduce meat consumption through replacement with meat-alternatives
- Assessment of putative psychosocial determinants of meat and meat-alternatives consumption will help to identify the active components of the intervention and will help inform future intervention development
- Health risk outcomes will provide preliminary evidence on potential health implications of replacing meat with meat-alternatives in the diet

Limitations

- Recruitment will occur among adult-only households within Oxford (UK), limiting the generalizability of the results
- The study will only provide proof of principle for the short-term effectiveness of a behavioural intervention to reduce meat consumption and future work will be needed to translate these insights into longer term-interventions in routine settings

For peer review only

For peer review only - http://bmjopen.bmj.com/site/about/guidelines.xhtml

Introduction

While meat is a source of important nutrients and can be part of a healthy diet (1), red and processed meat consumption is also associated with an increased risk of developing some forms of cancer (2–4), cardiovascular disease (5–8), and type-2-diabetes (7,9–11). Furthermore, producing meat can negatively affect the natural environment and contribute to anthropogenic global warming (12–14), which may also detrimentally affect human health (15–18). Reducing meat consumption could therefore help to promote public health and protect the natural environment, but a recent report identified "a remarkable lack of policies, initiatives or campaigns" designed to tackle the demand for meat (19). This state of inaction is partly due to the scarcity of evidence on the effectiveness of interventions to reduce meat consumption (19–23) warranting more experimental research to develop and evaluate such interventions. The rising availability of alternatives, such as textured vegetable proteins and mycoprotein-based alternatives (24) could help to reduce meat consumption, as these products resemble meat in their gastronomic function, appearance, and preparation. Nevertheless, uptake of meat-alternatives remains low in many developed countries (24–27), which might partly be due to a lack of familiarity with these foods (25,28,29). Interventions increasing people's familiarity with meat-alternatives could therefore help to overcome this barrier and, in turn, reduce meat consumption. A recent systematic review of experimental studies concluded that interventions that supplied plant-based alternatives were associated with reductions in meat consumption during, and several weeks after, the interventions (23). Nevertheless, this evidence is based on small uncontrolled pre-post intervention studies (30,31) and more systematic evaluations of the behavioural impact of such interventions is warranted. Additionally, there is currently no evidence from randomised trials on the psychosocial and health consequences of interventions aiming at reducing meat consumption through the replacement with meat-alternatives.

Objectives

The primary aim of the Replacing Meat with Alternative Plant-based products (RE-MAP) trial is to examine the effectiveness of a behavioural intervention to reduce meat consumption compared to a no intervention control condition. Additionally this study will evaluate the impact of the intervention on the consumption of other food groups, the nutritional composition of participants' diets, the putative psychosocial determinants of eating a low meat diet and of using plant-based meat-alternatives, and on biological markers of health risk, including blood lipid profiles, blood pressure, weight, and body composition. This study also aims to qualitatively investigate participants' experiences of the intervention, the mechanisms through which the intervention reduced meat consumption, and/or the barriers preventing the intervention to aid this dietary transition.

For peer review only - http://bmjopen.bmj.com/site/about/guidelines.xhtml

Methods

Study design and setting

The Re-MAP study will employ a two-arm parallel group individually randomised controlled trial to evaluate a four-week behavioural intervention to reduce meat consumption. The primary endpoint is defined as the change in average daily meat consumption at four weeks form baseline, assessed through self-reported seven days food diaries. The study will be conducted in Oxford, United Kingdom.

Recruitment

Participants will be recruited from the general population through advertisements in public buildings, newspapers, mailing lists, and social media. Individuals contacting the study team will receive a written information sheet summarizing the study protocol. Individuals confirming their interest will be called by the recruiting member of the research team, who will summarise the study protocol and answer any outstanding question. The recruiting member of the research team will also screen individuals against the eligibility criteria and invite eligible individuals to attend an enrolment appointment.

Eligibility criteria

Inclusion criteria:

(a) are ≥18 years old
(b) self-report to eat meat regularly
(c) belong to an adult-only household
(d) are willing to try meat-alternatives
(e) own adequate food storing facilities
(f) possess a device compatible with the requirements of the online food diary
(g) provide informed consent

Exclusion criteria:

(a) report they have relevant food allergies
(b) report suffering from an eating disorder
(c) report to be pregnant or plan pregnancy in the study period
(d) belong to the same household as a previously enrolled participant
(e) report consuming meat-alternatives more than once a week on average
(f) return baseline dietary records of insufficient quality for analysis
(g) the recruiting researcher deems the interested individual unable to adhere appropriately to the study protocol (e.g. insufficient knowledge of the English language, planned absences from main residence during the course of the study, enrolled in other longitudinal dietary intervention study).

For peer review only

For peer review only - http://bmjopen.bmj.com/site/about/guidelines.xhtml

Participant flow

Enrolment appointment

The enrolment appointment will take place on University premises. During this appointment an appropriately trained member of the research team will seek written informed consent (see supplementary file 1), witnessing this by means of dated signature. After gaining informed consent the enrolling member of the research team will set up participants' online food diaries to include six possible meal entries per day (breakfast, mid-morning, lunch, mid-afternoon, dinner, and post-dinner) and to allow the research team to remotely access participants' food diaries by means of a password. The recruiting member of the research team will also train participants in how to appropriately use the online food diaries and estimate portion sizes.

Baseline

Following the enrolment appointment, participants will complete a seven-day food diary over the week leading up to the following appointment, the baseline (T0). Participants not keeping sufficiently detailed diaries and those eating meat on less than five eating occasions over the week will be discontinued. At the baseline appointment an appropriately trained member of the research team will collect participants' food diaries, ask participants to answer the baseline online questionnaire, and measure participants blood lipids profile, blood pressure, weight, and body composition. At the end of the baseline appointment participants will be randomised to one of the two study conditions and will then follow the respective protocol for the next four weeks.

Follow up

Participants will be invited to attend a four-week (T1) and an eight-week (T2) follow-up and to keep a seven-days food diary over the week leading up to each follow-up. During the follow-up appointments a member of the research team will collect the respective food diary, ask participants to answer an online questionnaire, and measure participants blood lipids profile, blood pressure, weight, and body composition.

Sample size

Due to lack of research studies directly comparable to ours, pragmatic considerations have guided the decision to terminate recruitment once a sample of at least 100 volunteers have completed the four-weeks follow-up. A power analysis based on this pragmatically selected sample size suggests that 100 participants completing the primary outcome will allow detection of a medium effect size of d=0.6 with a power of 1-beta=0.84 and a two-tailed alpha criterion of 0.05.

Randomisation and blinding

Participants' group allocation will be based on a computer generated randomisation sequence, produced by an independent statistician. The randomisation sequence was designed to individually allocate participants to the intervention or control condition

For peer review only - http://bmjopen.bmj.com/site/about/guidelines.xhtml

in a 1:1 ratio and to achieve a proportional gender balance in the two conditions through blocking and stratification by sex. The research team is blinded to the randomisation sequence and to its block sizes and sequence. Allocation will be revealed to the researcher performing the randomisation only after the first food diary has been returned. Due to the nature of the intervention, participants and some members of the research team cannot be blind to participants' group allocation. The members of the research team analysing the food diaries will be blind to the group allocation. Due to the nature of the outcomes the risk of investigator bias will be low. To address the risk of social desirability bias in participants' reporting of foods intake and questionnaire responses, participants will be reminded during the enrolment visit and before each questionnaire that there are no right or wrong answers.

Intervention and comparator

Intervention

Re-MAP is a four-week behavioural intervention, which aims to reduce meat consumption, defined as non-seafood meat products, among regular meat eaters. Following an analysis of the target behaviour, i.e. a reduction in meat consumption, we included five psychosocial variables as the intervention's targets: attitudes, perceived behavioural control, and subjective social norms of eating a low meat diet, as well as attachment to meat, and eating identities (e.g. 'meat-eater' or 'vegetarian'). We then selected four intervention functions from the Behaviour Change Wheel (32,33) with the aim of influencing these psychosocial variables: (1) environmental restructuring enacted through providing meat-alternatives for four weeks, (2) training enacted through recipes, (3) education enacted through infographics on the health and environmental benefits of eating less meat, and (4) social modelling enacted through written vignettes outlining the story of people who reduced their meat consumption. These success stories were developed following an online patient and public involvement (PPI) activity. This PPI activity involved asking people who consciously reduced their consumption of meat to share their motives to do so, their strategies to enact this dietary transition, and the way they overcame the challenges associated with this transition. A logic model of the intervention is displayed in figure 1.

Insert here logic model

Figure 1: Intervention logic model

Comparator

Participants in the control condition will receive no intervention. The TiDIER checklist (34) for the Re-MAP intervention and the comparator is reported in table 1.

	Intervention	Comparator
BRIEF NAME	Re-MAP – a behavioural intervention to reduce meat consumption	No intervention
WHY	Environmental restructuring: Meat alternatives will be provided for one	N/A

For peer review only

For peer review only - http://bmjopen.bmj.com/site/about/guidelines.xhtml

	month with the aim of enhancing attitudes towards and behavioural control of eating a low meat diet by making meat-free alternative easily available to participants. This intervention component also aims to reduce participants' attachment to meat. Participants will select from a range of commercially available meat-alternatives including soy and other textured vegetable protein products (e.g. soy sausages), plant-based and pulses-based products (e.g. bean burgers), mycoprotein products (e.g. mycoprotein steaks). Meat alternatives will be defined as meat-free products that fulfil the same gastronomic function as products that normally contain meat (e.g. sausages, burgers, meatballs, steaks, or mince).<br><br>Training: Recipes will be provided with the aim of enhancing participants' behavioural control of eating a low meat diet by enhancing their skills of preparing meat-free meals.<br><br>Education: Information leaflets about the health and environmental benefits of eating less meat will be provided to enhance participants' attitudes towards eating a low meat diet and to reduce participants' attachment to meat.<br><br>Social modelling: written success stories of people who reduced their meat consumption will be provided to increase participants perceived social norm of eating a low meat diet and to promote the dietary identity of meat reducers, such as flexitarians.	
WHAT	Environmental restructuring: Participants will be provided with meat alternatives for one month, which they will be able to select from a printed catalogue of commercially available meat-alternatives. Participants will be asked to select enough meat-alternatives to have a meat-free product available on every occasion on which they would normally have meat for two weeks. Participants will be free to order enough foods to cater for themselves and other members of their household, if they wished to do so. The meat-alternatives will be delivered to participants' homes by a food retailer on up to two occasions over the intervention month: the first delivery will be scheduled immediately after participants are allocated to the intervention condition. The second delivery will be scheduled two weeks after the randomisation for participants who wish to top up their stock of meat-alternatives.<br><br>Training: A printed booklet containing 11 illustrated recipes of meat-alternatives will be delivered immediately after participants are allocated to the intervention condition. These recipes will incorporate some of the meat-alternatives used as part of this study. A second cookbook predominantly reporting on more general meat-free recipes (i.e. not focussing on meat alternatives) will be provided during the fourth intervention week. All participants received the same recipes.<br><br>Education: Participants will receive 8 printed pages of illustrated information on the health (4 pages) and environmental implications(4 pages) of eating less meat and 2 introduction pages and references delivered per post to their home over the course of the intervention month.. The info-graphics were developed using publicly available information from peer reviewed literature and relevant environmental or health organisations (e.g. cancer research UK). Immediately after being allocated to the intervention condition participants will receive an illustrated binder, which they will use to collect the information leaflets. The binder will include 2 pages of introductory information and the sources from which the information was drawn.<br><br>Success stories: Participants will receive three illustrated success stories vignettes delivered per post to their home during the last intervention week.	N/A

For peer review only - http://bmjopen.bmj.com/site/about/guidelines.xhtml

	The success stories will cover a range of different demographics (sex and age), different motives for eating less meat, and different strategies to transition to lower meat diets. The narratives will be about eating less meat rather than about ceasing to eat meat entirely. Participants will also receive a sheet on which they could report their own success story if the wish to do so. Participants will be asked to add this information to their illustrated binder.	
WHO	The lead researcher of this trial (FB) will deliver the intervention. An Access Database System will be used to schedule the deliveries of each intervention component ensuring that each intervention component will be delivered at the appropriate time for each participant.	N/A
HOW	The intervention consists in the delivery of the aforementioned materials. We will use the delivery services of one of UK's largest food retailers to purchase and deliver the meat-alternatives to participants. We will use Royal Mail to send printed materials. The binder will be delivered to participants immediately after they are randomised to the intervention condition.	N/A
WHERE	N/A	N/A
TAILORED	N/A	N/A
HOW Well	We elected to use a single study account with the food retailer to schedule all the study deliveries, which will enable us to monitor the successful completion and receipt of each delivery. Due to the nature of the intervention it will not be necessary to establish any other systems to monitor the fidelity of the intervention delivery.	N/A

Table 1: TiDIER checklist describing the Re-MAP intervention and no-intervention comparator

Patients and public involvement

Following the development of the basic intervention structure, we held a discussion group with ten members of the general public aiming to improve the acceptability and effectiveness of the RE-MAP intervention. We invited five meat eaters and five meat reducers to attend the discussion group, aiming to include people representing the target population of the intervention as well as people that successfully reduced their meat consumption. Public contributors were recruited using an established mailing list. The discussion group informed the development of each intervention component and of other aspects of the trial including:

- What type of meat-alternatives to offer as part of the intervention
- How to design the educational intervention components to be engaging and easily accessible to different publics
- What language to use as part of the success stories vignettes and how to increase their relatability
- What cookbooks and recipes to use as part of the intervention
- The likely burden of trial participation and how to best compensate trial participants

Contributors to the aforementioned public involvement activities will not be involved in other aspects of the trial implementation (such as recruitment) and will be asked not to enrol as trial participants, as they will have already reviewed much of the intervention material.

For peer review only - http://bmjopen.bmj.com/site/about/guidelines.xhtml

Outcomes

Primary outcome

- Change in mean daily grams of meat consumed between the baseline (T0) and the four-week follow-up (T1)

Secondary outcomes

- Change in mean daily grams of meat consumed between the baseline (T0) and the eight-week follow-up (T2)
- Change in the intention to eat a low meat diet between the baseline (T0) and both follow-up (T1, T2)
- Change in attachment to meat, eating identities, and in attitudes, perceived behavioural control, and subjective social norm of eating a low meat diet between the baseline (T0) and both follow-up (T1, T2)

Exploratory outcomes

- Change in participants' blood lipid profiles (total cholesterol, HDL cholesterol, triglycerides, LDL cholesterol, non-HDL cholesterol, LDL:HDL cholesterol ratio) between the baseline (T0) and both follow-up (T1, T2)
- Change in systolic and diastolic blood pressure between the baseline (T0) and both follow-up (T1, T2)
- Change in participants' body mass index between the baseline (T0) and both follow-up (T1, T2)
- Change in participants' body fat percentage between the baseline (T0) and both follow-up (T1, T2)
- Change in the number of meals containing foods from other food groups between the baseline (T0) and both follow-up (T1, T2)
- Change in participants' mean daily energy, macro-, and micronutrients intake between the baseline (T0) and both follow-up (T1, T2)
- Change in participants' intentions, attitudes, perceived behavioural control, and subjective social norms of using meat-alternatives between the baseline (T0) and both follow-up (T1, T2)
- Change in participants' desire for meat-substitutes to be similar to meat between the baseline (T0) and both follow-up (T1, T2)

Measurements

Table 2 provides a summary of the trial activities and of the measurement that will be collected at each stage of the trial.

	Visits				
	Telephone screening	Enrolment Visit	Baseline Visit	4 week follow up	8 week follow up
Enrolment					
Eligibility screening	X				
Informed consent		X			

For peer review only - http://bmjopen.bmj.com/site/about/guidelines.xhtml

Randomisation			X		
**Intervention**					
REMAP					
Control					
**Demographic and psychosocial traits**					
Demographics			X		
Food neophobia			X		
Self control scale			X		
**Dietary measurements**					
Food diary			X	X	X
Retrospective eating questionnaire			X	X	X
**Psychosocial variables**					
Attitude towards eating a low meat diet and using meat-alternatives			X	X	X
Perceived behavioural control of eating a low meat diet and using meat-alternatives			X	X	X
Subjective social norm of eating a low meat diet and using meat-alternatives			X	X	X
Intention to eat a low meat diet and to use meat-alternatives			X	X	X
Attachment to meat			X	X	X
Eating identity			X	X	X
Desire for similarity between meat and meat-alternatives			X	X	X
**Biophysical outcomes**					
Height			X		
Weight			X	X	X
Body composition			X	X	X
Blood pressure			X	X	X
Blood lipids profile			X	X	X
**Qualitative work-stream**					
Semi structured interviews					X

Table 2: Schedule of measurements and trial activities

Socio-demographic characteristics

- At the baseline, participants will be asked to self-report on their age, sex, highest degree, household income, household composition, ethnicity, and nationality

Psychological trait characteristics

- At the baseline, participants' trait food neophobia will be measured using a questionnaire scale adapted from Pliner and Hobden (35) including six items with a 7-point scale (disagree strongly – agree strongly)
- At the baseline, participants' self-control will also be assessed using a questionnaire scale adapted from Tangney et al. (36) including eight items with a 7-point scale (disagree strongly – agree strongly)

Dietary measurements

For peer review only - http://bmjopen.bmj.com/site/about/guidelines.xhtml

- Meat consumption will be measured in grams/day by disaggregating meat products recorded by participants on their seven-days food diaries. The daily average will exclude days in which energy intake was <1000kcal, which are considered unlikely to represent habitual consumption
- Average daily number of meals containing foods from other food groups will be measured counting the meals in participants' food diaries containing the food groups of interest, including:
    - Unprocessed pork meat
    - Unprocessed red meat from ruminants
    - Unprocessed poultry or game meat
    - Processed meat
    - Mycoprotein meat-alternatives
    - Soy-based meat-alternatives or meat-alternatives made of other textured vegetable protein
    - Other meat-alternatives (e.g. bean burgers)
    - Milk and yoghurt
    - Cheese
    - Dairy-free milk and yoghurt alternatives
    - Dairy-free cheese alternatives
    - Fish and seafood
    - Eggs
    - Pulses other than those in meat-alternatives
    - Vegetables other than those in meat-alternatives
    - Starchy foods other than those in meat-alternatives
    - Nuts and seeds other than those in meat-alternatives
    - Fruit
    - Savoury and sweet snacks
    - Soft drinks
    - Alcoholic drinks

  A retrospective eating questionnaire will also ask participants to recall the number of eating occasions on which they had the foods listed above over the week of their food diary. This questionnaire will only be used in sensitivity analyses
- The daily average energy intake and nutritional composition of participants' diets will be measured using data from the online food diary

Psychosocial variables

- Attachment to meat will be measured using the meat attachment questionnaire (37)
- Eating identities will be self-reported by participants among meat-eater, omnivore, flexitarian, pescatarian, vegetarian, vegan, or 'other'. The identities involving no consumption of non-seafood meat (i.e. pescatarian, vegetarian, and vegan) will be clustered together in non-meat eating identity

For peer review only - http://bmjopen.bmj.com/site/about/guidelines.xhtml

- Attitudes, subjective social norms, and perceived behavioural control to eat a low meat diet and to use meat-alternatives will be respectively assessed with three questionnaire items constructed following Francis et al. (38) on a 7-point scale (disagree strongly – agree strongly)
- Intentions to eat a low meat diet and to use meat-alternatives will be assessed using a single questionnaire item on a 7-point scale (disagree strongly – agree strongly)
- Desire for similarity between meat and meat-alternatives will be assessed using 11 questionnaire items with a 7-point scale (disagree strongly – agree strongly) adapted from Hoek et al. (25)

Physical measures

- Blood lipids profiles (total cholesterol, HDL cholesterol, triglycerides, LDL cholesterol, non-HDL cholesterol, LDL:HDL cholesterol ratio) will be measured using Alere Cholestech LDX®
- Height will be measured to the nearest 0.1cm using a stadiometer
- Weight and body composition will be measured using an electronic scale (SC-240 MA, Tanita Japan), which records the proportion of body fat using bioelectrical impedance. Weight was recorded to the nearest 0.1kg
- Seated blood pressure will be measured as the average of the second and third reading of three seated readings

Retention

We will use reminder text messages to increase attendance to each of the four study appointments. Additionally participants will receive financial compensation for partaking in each of the 3 assessment visits. Participants will have the right to withdraw from the study at any time. The principal investigator will have the right to discontinue participants' involvement in the study when they become ineligible and/or when significant protocol deviations occur. The data of participants who withdraw will be kept and might be used in exploratory and sensitivity analyses, unless the participant requests for the data to be deleted.

Adverse events

Any study-related adverse event will be reported to the Research Ethics Committee in accordance to Good Clinical Practice (GCP). All study-related adverse events will be included in the final trial report.

Data management

Data will be entered by a trained member of the research team and stored in an OpenClinica database that was specifically developed for this trial. The database will feature ranges and validation checks to promote reliability in the data entry process. Data recording and storage will run in accordance with GCP.

Statistical analyses

For peer review only - http://bmjopen.bmj.com/site/about/guidelines.xhtml

We will employ linear models to investigate whether changes in meat consumption between the baseline and both follow-up differ significantly between the intervention and the control group. Our main analysis will employ unadjusted models and only include data from participants completing the relevant follow-up. Sensitivity analysis will be performed with a Baseline Observation Carried Forward (BOCF) assumption for missing data and adjusting for baseline variables. The intervention effect will be reported with 95% CI and p-values. A two-tailed criterion p-value of alpha=0.05 will be used to assess the statistical significance of the results. The same procedure will be employed to assess whether changes in the other pre-specified dietary, nutritional, psychosocial, and biophysical outcomes between the baseline and both follow-up differ significantly between the control and the intervention group. Detailed main-, subgroup-, and sensitivity analyses plans will be finalised before conducting any specific outcome analysis. No interim analysis is planned.

Qualitative study

After the eight-week follow-up, a subsample of participants receiving the intervention will be invited to take part in a semi-structured interview. This qualitative study aims to understand participants' experiences of the intervention, the mechanisms through which the intervention helped reducing meat consumption, or the barriers preventing the intervention to aid this transition. The semi-structured interviews will follow a discussion guide while also remaining sensitive to unsolicited themes. The interview will set the context by asking participants to elaborate on their motivation to volunteer for the trial and on their thoughts and feelings towards reducing meat consumption prior to enrolling into the study. Participants will then be encouraged to elaborate on the mechanisms through which they felt that the intervention helped them eat less meat or the barriers preventing the intervention to do so. In doing so participants will be prompted to think about the intervention in its entirety as well as about each individual intervention component. Participants will be encouraged to elaborate on their perceived ability and motivation to maintain a lower consumption of meat after the intervention period and beyond the context of the study. Whenever possible we will use open questions to encourage participants to elaborate on their thoughts and feelings freely and in depth. We aim to avoid questions of evaluative nature to minimise the risk of social desirability bias. We anticipate interviewing 20 participants, however sampling will be extended should new themes emerge during the interviewing process. We will employ a purposeful sampling technique aiming to achieve a sex balance. Participants will be free to decide whether or not to be interviewed. No additional compensation will be offered to participants agreeing to be interviewed. Qualitative interviews will be conducted in person and transcribed verbatim. Transcriptions will be analysed using NVIVO and employing a data driven thematic analysis to identify codes and to group these codes into broader themes.

Trial steering committee

The principal investigator will be responsible for the project coordination and the senior investigators will oversee the operational aspects of the trial. The authors of

For peer review only - http://bmjopen.bmj.com/site/about/guidelines.xhtml

this protocol will form the trial management group (TMG), which will regularly monitor the study implementation, as well as the data generation, documentation, and reporting. All members of the TMG are trained in GCP and will take appropriate actions to safeguard participants and the quality of the trial. Access to data will be granted to appropriate members of the research team and to authorised representatives from the host institution to monitor and/or audit the study and ensure compliance with regulations.

Ethics and dissemination

The investigators will ensure that this study is conducted in accordance with the principles of the Declaration of Helsinki, with relevant institutional regulations, with GCP, and GDPR regulations. This study was reviewed and received ethical approval by the Medical Sciences Interdivisional Research Ethics Committee of the University of Oxford (R54329/RE001). Substantial planned changes to the protocol, an end of study notification, and a final report will be submitted to the aforementioned research ethics committee. The results of this RCT will be reported following the Consolidated Standards of Reporting Trials guidelines (39) and submitted for publication to scientific journals, regardless of the outcome. Authorship will be determined in accordance with the ICMJE guidelines. Contributors of other parties and funding will be acknowledged. Results will also be presented at national and international conferences and disseminated through established networks. A lay summary will be distributed through an established newsletter to which participants can subscribe on their last study appointment.

Sponsor

University of Oxford
University Offices
Wellington Square
Oxford
OX1 2JD
United Kingdom

The sponsor has no involvement in the implementation of the study.

Acknowledgements

We thank all the PPI contributors for having helped us develop the RE-MAP intervention. We thank Lynne Maddocks for her assistance in forming the PPI panel for this study. We thank Lucy Eldridge for her support in developing the study database. We thank Jason Oke for his assistance in developing the randomization sequence. We thank Alexa Hayley and Bernhard Haring for their comments on previous versions of this manuscript.

Authors' contributions: All authors have been involved in shaping each stage of this research protocol. FB has written this protocol and developed the intervention and led on the study design. FB, SAJ, and PA have designed the study. FB and NA

For peer review only - http://bmjopen.bmj.com/site/about/guidelines.xhtml

have developed the trial management system. NA, BC, and EC have contributed in designing this research and the intervention.

Funding: This research is funded by the Wellcome Trust, Our Planet Our Health programme (Livestock, Environment and People - LEAP), award number 205212/Z/16/Z. FB's time on this project is funded by the Medical Research Council (MRC), Green Templeton College Oxford, and the National Institute for Health Research (NIHR) School for Primary Care Research (SPCR). EC's and BC's time on this project is funded by the Wellcome Trust, Our Planet Our Health programme (Livestock, Environment and People - LEAP), award number 205212/Z/16/Z. NA, PA, and SAJ are supported by the NIHR Oxford Biomedical Research Centre and Collaboration for Leadership in Applied Health Research and Care Oxford at Oxford Health NHS Foundation Trust. PA and SAJ are NIHR Senior Investigators.

Competing interests statement

The authors have no known competing interests to declare.

References

1. Scarborough P, Kaur A, Cobiac L, Owens P. Eatwell Guide: modelling the dietary and cost implications of incorporating new sugar and fibre guidelines. BMJ Open [Internet]. 2016 [cited 2017 May 31]; Available from: http://bmjopen.bmj.com/content/6/12/e013182.abstract
2. Bouvard V, Loomis D, Guyton KZ, Grosse Y, Ghissassi F El, Benbrahim-Tallaa L, et al. Carcinogenicity of consumption of red and processed meat. Lancet Oncol [Internet]. Elsevier; 2015 Dec 1 [cited 2017 Nov 9];16(16):1599–600. Available from: http://linkinghub.elsevier.com/retrieve/pii/S1470204515004441
3. Chan DSM, Lau R, Aune D, Vieira R, Greenwood DC, Kampman E, et al. Red and processed meat and colorectal cancer incidence: meta-analysis of prospective studies. PLoS One [Internet]. Public Library of Science; 2011 Jan 6 [cited 2015 Oct 26];6(6):e20456. Available from: http://journals.plos.org/plosone/article?id=10.1371/journal.pone.0020456
4. Parkin DM, Boyd L, Walker LC. 16. The fraction of cancer attributable to lifestyle and environmental factors in the UK in 2010. Br J Cancer [Internet]. 2011 Dec 6 [cited 2014 Oct 30];105 Suppl:S77-81. Available from: http://www.pubmedcentral.nih.gov/articlerender.fcgi?artid=3252065&tool=pmcentrez&rendertype=abstract
5. Schwingshackl L, Hoffmann G, Lampousi A-M, Knüppel S, Iqbal K,

For peer review only - http://bmjopen.bmj.com/site/about/guidelines.xhtml

Schwedhelm C, et al. Food groups and risk of type 2 diabetes mellitus: a systematic review and meta-analysis of prospective studies. Eur J Epidemiol [Internet]. Springer Netherlands; 2017 Apr 10 [cited 2017 May 23];1–13. Available from: http://link.springer.com/10.1007/s10654-017-0246-y

6. Micha R, Wallace SK, Mozaffarian D. Red and Processed Meat Consumption and Risk of Incident Coronary Heart Disease, Stroke, and Diabetes Mellitus: A Systematic Review and Meta-Analysis. Circulation [Internet]. 2010 Jun 1 [cited 2017 May 23];121(21):2271–83. Available from: http://www.ncbi.nlm.nih.gov/pubmed/20479151

7. Micha R, Michas G, Mozaffarian D. Unprocessed red and processed meats and risk of coronary artery disease and type 2 diabetes--an updated review of the evidence. Curr Atheroscler Rep [Internet]. 2012 Dec [cited 2015 Dec 27];14(6):515–24. Available from: http://www.pubmedcentral.nih.gov/articlerender.fcgi?artid=3483430&tool=pmcentrez&rendertype=abstract

8. Song M, Fung TT, Hu FB, Willett WC, Longo VD, Chan AT, et al. Association of Animal and Plant Protein Intake With All-Cause and Cause-Specific Mortality. JAMA Intern Med [Internet]. 2016 Oct 1 [cited 2018 Dec 6];176(10):1453. Available from: http://www.ncbi.nlm.nih.gov/pubmed/27479196

9. Feskens EJM, Sluik D, van Woudenbergh GJ. Meat Consumption, Diabetes, and Its Complications. Curr Diab Rep [Internet]. 2013 Apr 25 [cited 2017 Nov 9];13(2):298–306. Available from: http://www.ncbi.nlm.nih.gov/pubmed/23354681

10. Barnard N, Levin S, Trapp C. Meat consumption as a risk factor for type 2 diabetes. Nutrients [Internet]. Multidisciplinary Digital Publishing Institute (MDPI); 2014 Feb 21 [cited 2017 Nov 9];6(2):897–910. Available from: http://www.ncbi.nlm.nih.gov/pubmed/24566443

11. Pan A, Sun Q, Bernstein AM, Manson JE, Willett WC, Hu FB. Changes in Red Meat Consumption and Subsequent Risk of Type 2 Diabetes Mellitus. JAMA Intern Med [Internet]. 2013 Jul 22 [cited 2018 Dec 6];173(14):1328. Available from: http://www.ncbi.nlm.nih.gov/pubmed/23779232

12. Steinfeld H, Gerber P, Wassenaar T. Livestock's long shadow. Issues Options, … [Internet]. 2006 [cited 2017 May 23]; Available from: https://www.rfp-europe.org/fileadmin/SITE_ERFP/ERFP_meetings/2007_Dublin/Dublin2

For peer review only - http://bmjopen.bmj.com/site/about/guidelines.xhtml

007_LivestocksLongShadow_HOFFMANN.pdf

13. Pimentel D, Pimentel M. Sustainability of meat-based and plant-based diets and the environment. Am J Clin Nutr [Internet]. American Society for Nutrition; 2003 Sep [cited 2017 May 23];78(3 Suppl):660S–663S. Available from: http://www.ncbi.nlm.nih.gov/pubmed/12936963

14. Tilman D, Clark M. Global diets link environmental sustainability and human health. Nature [Internet]. Nature Publishing Group, a division of Macmillan Publishers Limited. All Rights Reserved.; 2014 Nov 27 [cited 2015 Jul 27];515(7528):518–22. Available from: http://dx.doi.org/10.1038/nature13959

15. Watts N, Amann M, Ayeb-Karlsson S, Belesova K, Bouley T, Boykoff M, et al. The Lancet Countdown on health and climate change: from 25 years of inaction to a global transformation for public health. Lancet [Internet]. Elsevier; 2017 Oct 30 [cited 2018 Jan 25]; Available from: https://www.sciencedirect.com/science/article/pii/S0140673617324649?_rdoc=1&_fmt=high&_origin=gateway&_docanchor=&md5=b8429449ccfc9c30159a5f9aeaa92ffb

16. Mcmichael AJ, Campbell-Lendrum DH, Corvalán CF, Ebi KL, Githeko AK, Scheraga JD, et al. Climate change and human health. Risks and responses [Internet]. 2003 [cited 2018 Jan 25]. Available from: http://www.who.int/globalchange/publications/climchange.pdf

17. Economou V, Gousia P. Agriculture and food animals as a source of antimicrobial-resistant bacteria. Infect Drug Resist [Internet]. Dove Press; 2015 [cited 2018 Jan 25];8:49–61. Available from: http://www.ncbi.nlm.nih.gov/pubmed/25878509

18. Tang KL, Caffrey NP, Nóbrega DB, Cork SC, Ronksley PE, Barkema HW, et al. Restricting the use of antibiotics in food-producing animals and its associations with antibiotic resistance in food-producing animals and human beings: a systematic review and meta-analysis. Lancet Planet Heal [Internet]. Elsevier; 2017 Nov 1 [cited 2018 May 13];1(8):e316–27. Available from: http://www.ncbi.nlm.nih.gov/pubmed/29387833

19. Bailey R, Froggatt A WL. Livestock–climate change's forgotten sector. [Internet]. 2014 [cited 2017 Nov 10]. Available from: https://scholar.google.co.uk/scholar?hl=en&as_sdt=0%2C5&q=Livestock+–

For peer review only - http://bmjopen.bmj.com/site/about/guidelines.xhtml

+Climate+Change's+Forgotten+Sector+Global+Public+Opinion+on+Meat+and+Dairy+Consumption&btnG=

20. Garnett T, Mathewson S, Angelides P, Borthwick F. Policies and actions to shift eating patterns: What works? [Internet]. 2015 [cited 2017 May 24]. Available from: http://www.fcrn.org.uk/sites/default/files/fcrn_chatham_house_0.pdf
21. Wellesley L, Happer C, Froggatt A. Chatham House Report Changing Climate, Changing Diets Pathways to Lower Meat Consumption. 2015 [cited 2017 Nov 10]; Available from: https://www.chathamhouse.org/sites/files/chathamhouse/publications/research/CHHJ3820 Diet and climate change 18.11.15_WEB_NEW.pdf
22. Bianchi F, Dorsel C, Garnett E, Aveyard P, Jebb SA. Interventions targeting conscious determinants of human behaviour to reduce the demand for meat: a systematic review with qualitative comparative analysis. Int J Behav Nutr Phys Act [Internet]. BioMed Central; 2018 Dec 19 [cited 2018 Dec 6];15(1):102. Available from: https://ijbnpa.biomedcentral.com/articles/10.1186/s12966-018-0729-6
23. Bianchi F, Garnett E, Dorsel C, Aveyard P, Jebb SA. Restructuring physical micro-environments to reduce the demand for meat: a systematic review and qualitative comparative analysis. Lancet Planet Heal [Internet]. Elsevier; 2018 Sep 1 [cited 2018 Dec 6];2(9):e384–97. Available from: http://www.ncbi.nlm.nih.gov/pubmed/30177007
24. Mintel Group Ltd. Meat-free Foods - UK [Internet]. 2017 [cited 2017 Nov 12]. Available from: http://academic.mintel.com/sinatra/oxygen_academic/list/id=796253&type=RCItem#0_1___page_RCItem=0
25. Hoek A, Luning P, Weijzen P, Engels W, Kok F. Replacement of meat by meat substitutes. A survey on person-and product-related factors in consumer acceptance. Appetite [Internet]. 2011 [cited 2017 May 24]; Available from: http://www.sciencedirect.com/science/article/pii/S0195666311000523
26. Hartmann C, Siegrist M. Consumer perception and behaviour regarding sustainable protein consumption: A systematic review. Trends Food Sci Technol [Internet]. 2017 Mar [cited 2017 Nov 26];61(61):11–25. Available from: http://linkinghub.elsevier.com/retrieve/pii/S0924224416302904
27. Kumar P, Chatli MK, Mehta N, Singh P, Malav OP, Verma AK. Critical

For peer review only - http://bmjopen.bmj.com/site/about/guidelines.xhtml

Reviews in Food Science and Nutrition Meat analogues: Health promising sustainable meat substitutes Meat analogues: Health promising sustainable meat substitutes. 2017 [cited 2018 May 16]; Available from: http://www.tandfonline.com/action/journalInformation?journalCode=bfsn20

28. Clery E, Bailey R. Food technologies Findings from the 2008 British Social Attitudes survey. Food Technol Find from 2008 Br [Internet]. 2010 [cited 2017 May 24]; Available from: http://www.fcrn.org.uk/sites/default/files/BSA_Food_technologies_findings.pdf

29. Hoek AC, Elzerman JE, Hageman R, Kok FJ, Luning PA, Graaf C de. Are meat substitutes liked better over time? A repeated in-home use test with meat substitutes or meat in meals. Food Qual Prefer [Internet]. Elsevier; 2013 Apr 1 [cited 2018 May 13];28(1):253–63. Available from: https://www.sciencedirect.com/science/article/abs/pii/S0950329312001280

30. Clark M. Chronic Effects Of Replacing Red And Processed Meat With Non/Reduced Meat Alternatives [Internet]. Cambridge University Press; 2017 [cited 2017 Nov 12]. Available from: https://www.cambridge.org/core/product/identifier/S0029665117001434/type/journal_article

31. Holloway T, Salter AM, McCullough FS. Dietary intervention to reduce meat intake by 50% in University students – a pilot study. Proc Nutr Soc [Internet]. Cambridge University Press; 2012 Jan 19 [cited 2017 Nov 12];71(OCE2):E164. Available from: http://www.journals.cambridge.org/abstract_S0029665112002212

32. Michie S, Atkins L, West R. The behaviour change wheel: a guide to designing interventions. Needed: physician leaders [Internet]. 2014 [cited 2017 May 24]; Available from: http://www.physicianleaders.ca/assets/cspljournalsummer2015.pdf#page=26

33. Michie S, Stralen M van. The behaviour change wheel: a new method for characterising and designing behaviour change interventions. 2011 [cited 2017 May 24]; Available from: https://implementationscience.biomedcentral.com/articles/10.1186/1748-5908-6-42

For peer review only - http://bmjopen.bmj.com/site/about/guidelines.xhtml

34. Hoffmann TC, Glasziou PP, Boutron I, Milne R, Perera R, Moher D, et al. Better reporting of interventions: template for intervention description and replication (TIDieR) checklist and guide. BMJ [Internet]. 2014 [cited 2017 Sep 1];348. Available from: http://www.bmj.com/content/348/bmj.g1687

35. Pliner P, Hobden K. Development of a scale to measure the trait of food neophobia in humans. Appetite [Internet]. 1992 Oct [cited 2018 May 13];19(2):105–20. Available from: http://www.ncbi.nlm.nih.gov/pubmed/1489209

36. Tangney J, … AB-S-R and, 2018 undefined. High self-control predicts good adjustment, less pathology, better grades, and interpersonal success. taylorfrancis.com [Internet]. [cited 2018 May 13]; Available from: https://www.taylorfrancis.com/books/e/9781351707756/chapters/10.4324%2F9781315175775-12

37. Graça J, Calheiros MM, Oliveira A. Attached to meat? (Un)Willingness and intentions to adopt a more plant-based diet. Appetite [Internet]. 2015 Dec [cited 2017 Nov 13];95:113–25. Available from: http://www.ncbi.nlm.nih.gov/pubmed/26148456

38. Francis J, Eccles M, Johnston M, Walker A. Constructing questionnaires based on the theory of planned behaviour: A manual for health services researchers. 2004 [cited 2018 May 13]; Available from: http://openaccess.city.ac.uk/1735/1/TPB Manual FINAL May2004.pdf

39. Schulz KF, Altman DG, Moher D. CONSORT 2010 Statement: updated guidelines for reporting parallel group randomised trials. BMC Med [Internet]. 2010 Jan [cited 2015 Mar 14];8(1):18. Available from: http://www.biomedcentral.com/1741-7015/8/18

For peer review only

For peer review only - http://bmjopen.bmj.com/site/about/guidelines.xhtml

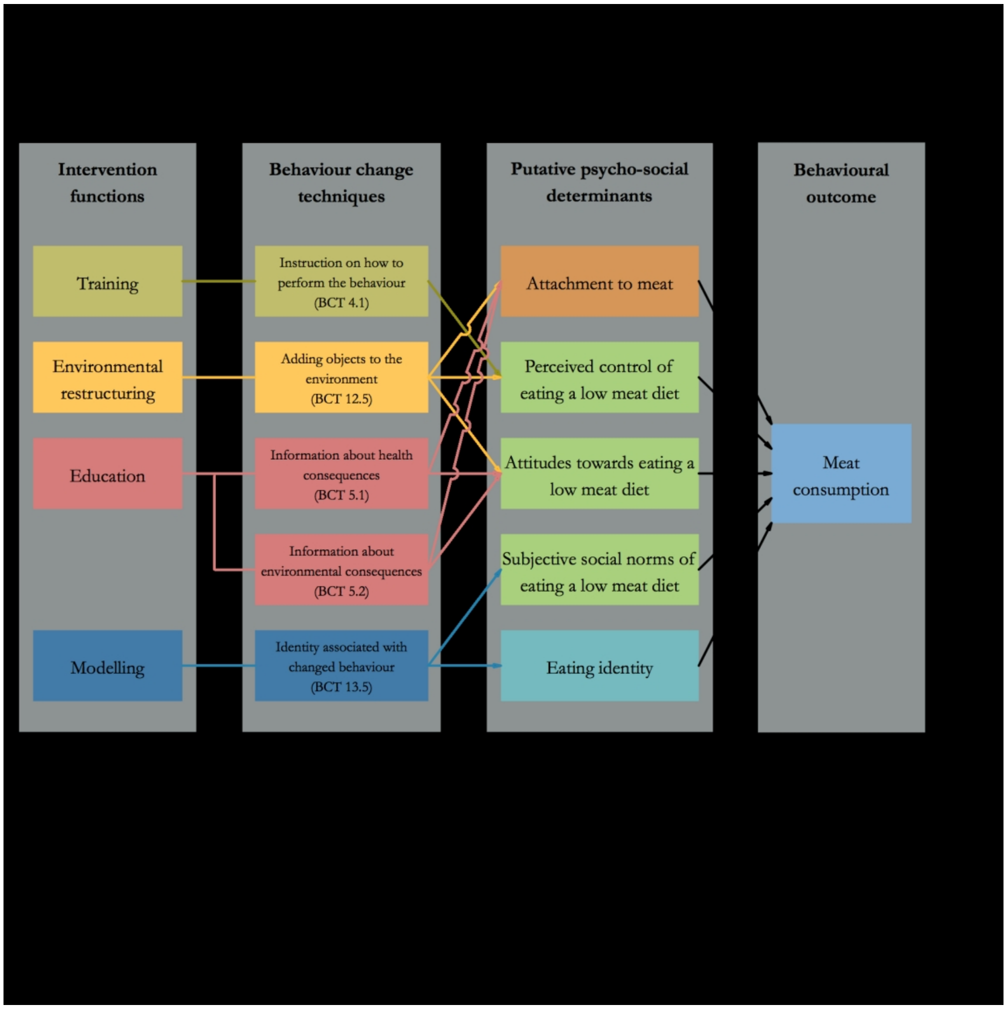
Intervention functions
Behaviour change techniques
Putative psycho-social determinants
Behavioural outcome
Training
Environmental restructuring
Education
Modelling
Instruction on how to perform the behaviour (BCT 4.1)
Adding objects to the environment (BCT 12.5)
Information about health consequences (BCT 5.1)
Information about environmental consequences (BCT 5.2)
Identity associated with changed behaviour (BCT 13.5)
Attachment to meat
Perceived control of eating a low meat diet
Attitudes towards eating a low meat diet
Subjective social norms of eating a low meat diet
Eating identity
Meat consumption

NUFFIELD DEPARTMENT OF PRIMARY CARE HEALTH SCIENCES

Principal Researcher: Filippo Bianchi
Contact Details: RE-MAP@phc.ox.ac.uk
Departmental web page: www.phc.ox.ac.uk
Department's address: Nuffield Department of Primary Care health Sciences, Radcliffe Observatory Quarter, Woodstock Road, Oxford. OX2 6GG
University web page: www.ox.ac.uk

Participant ID: ________________________________

Replacing **M**eat with **A**lternative **P**rotein **S**ources (RE-MAP STUDY)

Participant Consent Form. CUREC-Approval Reference: R54329/RE001

The purpose of this study is to test the effectiveness of a behavioural intervention designed to help people reduce their consumption of meat.

Please initial the boxes to confirm you agree

I confirm that I have read and understood the information sheet for the above study. I have had the opportunity to consider the information, ask questions and have had these answered satisfactorily.	☐
I understand that my participation is voluntary and that I am free to withdraw at any time, without having to give any reason, and without any adverse consequences.	☐
I understand that designated individuals may look at research data collected during the study where it is relevant to my taking part in this study. I give permission for these individuals to access my data.	☐
I understand that this project has been reviewed by, and received ethics clearance through, the University of Oxford Central University Research Ethics Committee.	☐
I understand who will have access to personal data provided, how the data will be stored, and what will happen to the data at the end of the project.	☐
I understand that three finger prick blood samples will be taken from me during this study to measure blood cholesterol. I understand the procedure that will be used to carry out these analyses.	☐
I understand this research will be written up as a student's thesis, I understand how personal data included in that thesis will be published and stored.	☐

For peer review only - http://bmjopen.bmj.com/site/about/guidelines.xhtml

I understand that I may be quoted in an anonymous way in publications pertinent to this study and that I will *not* be identified personally in any of these publications.	☐
I understand how to raise a concern or make a complaint.	☐
I consent to being audio recorded.	☐
I understand how audio recordings will be used in research outputs	☐
I consent to take part in the above study.	☐
Optional: Should I be allocated to the intervention group, I understand and consent for my name, address, telephone number, and selection of meat substitutes to be shared with Sainsbury's to carry out the food deliveries.	☐
Optional: I agree for research data collected in this study to be given to researchers, including those working outside of the EU, to be used in other research studies. I understand that any data that leave the research group will be fully anonymised so that I cannot be identified.	☐

Name of participant ______ Date ______ Signature ______

Name of person taking consent ______ Date ______ Signature ______

For peer review only - http://bmjopen.bmj.com/site/about/guidelines.xhtml

SPIRIT

STANDARD PROTOCOL ITEMS: RECOMMENDATIONS FOR INTERVENTIONAL TRIALS

SPIRIT 2013 Checklist: Recommended items to address in a clinical trial protocol and related documents*

Section/item	Item No	Description
**Administrative information**		
Title	1	Descriptive title identifying the study design, population, interventions, and, if applicable, trial acronym<br>P.1
Trial registration	2a	Trial identifier and registry name. If not yet registered, name of intended registry<br>P.1
	2b	All items from the World Health Organization Trial Registration Data Set<br>Provided in the protocol and in the ISRCTN trial registration (http://www.isrctn.com/ISRCTN13180635?q=filippo%20bianchi&filters=&sort=&offset=1&totalResults=1&page=1&pageSize=10&searchType=basic-search)
Protocol version	3	Date and version identifier<br>**Date applied**<br>22/06/2018<br>**Registered Online**<br>25/06/2018<br>**Published**<br>tbc
Funding	4	Sources and types of financial, material, and other support<br>P.14
Roles and responsibilities	5a	Names, affiliations, and roles of protocol contributors<br>P.1, P.14
	5b	Name and contact information for the trial sponsor<br>P.14
	5c	Role of study sponsor and funders, if any, in study design; collection, management, analysis, and interpretation of data; writing of the report; and the decision to submit the report for publication, including whether they will have ultimate authority over any of these activities<br>P.14

For peer review only - http://bmjopen.bmj.com/site/about/guidelines.xhtml

	5d	Composition, roles, and responsibilities of the coordinating centre, steering committee, endpoint adjudication committee, data management team, and other individuals or groups overseeing the trial, if applicable (see Item 21a for data monitoring committee)<br>P.13
**Introduction**		
Background and rationale	6a	Description of research question and justification for undertaking the trial, including summary of relevant studies (published and unpublished) examining benefits and harms for each intervention<br>P.3
	6b	Explanation for choice of comparators<br>P.3
Objectives	7	Specific objectives or hypotheses<br>P.3
Trial design	8	Description of trial design including type of trial (eg, parallel group, crossover, factorial, single group), allocation ratio, and framework (eg, superiority, equivalence, noninferiority, exploratory)<br>P.4
**Methods: Participants, interventions, and outcomes**		
Study setting	9	Description of study settings (eg, community clinic, academic hospital) and list of countries where data will be collected. Reference to where list of study sites can be obtained<br>P.4
Eligibility criteria	10	Inclusion and exclusion criteria for participants. If applicable, eligibility criteria for study centres and individuals who will perform the interventions (eg, surgeons, psychotherapists)<br>P.4
Interventions	11a	Interventions for each group with sufficient detail to allow replication, including how and when they will be administered<br>P. 6-8
	11b	Criteria for discontinuing or modifying allocated interventions for a given trial participant (eg, drug dose change in response to harms, participant request, or improving/worsening disease)<br>N/A

For peer review only

	11c	Strategies to improve adherence to intervention protocols, and any procedures for monitoring adherence (eg, drug tablet return, laboratory tests)<br>P.12
	11d	Relevant concomitant care and interventions that are permitted or prohibited during the trial<br>P.4
Outcomes	12	Primary, secondary, and other outcomes, including the specific measurement variable (eg, systolic blood pressure), analysis metric (eg, change from baseline, final value, time to event), method of aggregation (eg, median, proportion), and time point for each outcome. Explanation of the clinical relevance of chosen efficacy and harm outcomes is strongly recommended<br>P. 8-12
Participant timeline	13	Time schedule of enrolment, interventions (including any run-ins and washouts), assessments, and visits for participants. A schematic diagram is highly recommended (see Figure)<br>P. 9-10
Sample size	14	Estimated number of participants needed to achieve study objectives and how it was determined, including clinical and statistical assumptions supporting any sample size calculations<br>P. 5
Recruitment	15	Strategies for achieving adequate participant enrolment to reach target sample size<br>P.4

Methods: Assignment of interventions (for controlled trials)

Allocation:

Sequence generation	16a	Method of generating the allocation sequence (eg, computer-generated random numbers), and list of any factors for stratification. To reduce predictability of a random sequence, details of any planned restriction (eg, blocking) should be provided in a separate document that is unavailable to those who enrol participants or assign interventions<br>P. 5-6
Allocation concealment mechanism	16b	Mechanism of implementing the allocation sequence (eg, central telephone; sequentially numbered, opaque, sealed envelopes), describing any steps to conceal the sequence until interventions are assigned<br>P. 5-6

Implementation	16c	Who will generate the allocation sequence, who will enrol participants, and who will assign participants to interventions<br>P. 5-6
Blinding (masking)	17a	Who will be blinded after assignment to interventions (eg, trial participants, care providers, outcome assessors, data analysts), and how<br>P. 5-6
	17b	If blinded, circumstances under which unblinding is permissible, and procedure for revealing a participant's allocated intervention during the trial<br>P. 5-6

Methods: Data collection, management, and analysis

Data collection methods	18a	Plans for assessment and collection of outcome, baseline, and other trial data, including any related processes to promote data quality (eg, duplicate measurements, training of assessors) and a description of study instruments (eg, questionnaires, laboratory tests) along with their reliability and validity, if known. Reference to where data collection forms can be found, if not in the protocol<br>P. 5, P. 9-12
	18b	Plans to promote participant retention and complete follow-up, including list of any outcome data to be collected for participants who discontinue or deviate from intervention protocols<br>P. 12
Data management	19	Plans for data entry, coding, security, and storage, including any related processes to promote data quality (eg, double data entry; range checks for data values). Reference to where details of data management procedures can be found, if not in the protocol<br>P. 12
Statistical methods	20a	Statistical methods for analysing primary and secondary outcomes. Reference to where other details of the statistical analysis plan can be found, if not in the protocol<br>P. 12
	20b	Methods for any additional analyses (eg, subgroup and adjusted analyses)<br>P. 12
	20c	Definition of analysis population relating to protocol non-adherence (eg, as randomised analysis), and any statistical methods to handle missing data (eg, multiple imputation)<br>P. 12

Methods: Monitoring

Data monitoring	21a	Composition of data monitoring committee (DMC); summary of its role and reporting structure; statement of whether it is independent from the sponsor and competing interests; and reference to where further details about its charter can be found, if not in the protocol. Alternatively, an explanation of why a DMC is not needed<br>P.13
	21b	Description of any interim analyses and stopping guidelines, including who will have access to these interim results and make the final decision to terminate the trial<br>P.12
Harms	22	Plans for collecting, assessing, reporting, and managing solicited and spontaneously reported adverse events and other unintended effects of trial interventions or trial conduct<br>P.12
Auditing	23	Frequency and procedures for auditing trial conduct, if any, and whether the process will be independent from investigators and the sponsor<br>P.13
**Ethics and dissemination**		
Research ethics approval	24	Plans for seeking research ethics committee/institutional review board (REC/IRB) approval<br>P.13
Protocol amendments	25	Plans for communicating important protocol modifications (eg, changes to eligibility criteria, outcomes, analyses) to relevant parties (eg, investigators, REC/IRBs, trial participants, trial registries, journals, regulators)<br>P.14
Consent or assent	26a	Who will obtain informed consent or assent from potential trial participants or authorised surrogates, and how (see Item 32)<br>P. 5
	26b	Additional consent provisions for collection and use of participant data and biological specimens in ancillary studies, if applicable<br>N/A
Confidentiality	27	How personal information about potential and enrolled participants will be collected, shared, and maintained in order to protect confidentiality before, during, and after the trial<br>P.12, 13
Declaration of interests	28	Financial and other competing interests for principal investigators for the overall trial and each study site<br>P. 15

For peer review only - http://bmjopen.bmj.com/site/about/guidelines.xhtml

Access to data	29	Statement of who will have access to the final trial dataset, and disclosure of contractual agreements that limit such access for investigators<br>P. 13
Ancillary and post-trial care	30	Provisions, if any, for ancillary and post-trial care, and for compensation to those who suffer harm from trial participation<br>N/A
Dissemination policy	31a	Plans for investigators and sponsor to communicate trial results to participants, healthcare professionals, the public, and other relevant groups (eg, via publication, reporting in results databases, or other data sharing arrangements), including any publication restrictions<br>P. 14
	31b	Authorship eligibility guidelines and any intended use of professional writers<br>P. 13-14
	31c	Plans, if any, for granting public access to the full protocol, participant-level dataset, and statistical code<br>Data will be provided on reasonable requests.
**Appendices**		
Informed consent materials	32	Model consent form and other related documentation given to participants and authorised surrogates<br>Attached
Biological specimens	33	Plans for collection, laboratory evaluation, and storage of biological specimens for genetic or molecular analysis in the current trial and for future use in ancillary studies, if applicable<br>N/A

*It is strongly recommended that this checklist be read in conjunction with the SPIRIT 2013 Explanation & Elaboration for important clarification on the items. Amendments to the protocol should be tracked and dated. The SPIRIT checklist is copyrighted by the SPIRIT Group under the Creative Commons "Attribution-NonCommercial-NoDerivs 3.0 Unported" license.

For peer review only - http://bmjopen.bmj.com/site/about/guidelines.xhtml

Replacing meat with alternative plant-based products (RE-MAPs): Protocol for a randomized controlled trial of a behavioural intervention to reduce meat consumption

Journal:	*BMJ Open*
Manuscript ID	bmjopen-2018-027016.R2
Article Type:	Protocol
Date Submitted by the Author:	20-Mar-2019
Complete List of Authors:	Bianchi, Filippo; University of Oxford, Nuffield Department of Primary Care Health Science<br>Aveyard, Paul; University of Oxford, Primary Care Health Sciences<br>Astbury, Nerys; University of Oxford, Nuffiled Department of Primary Care Health Sciences<br>Cook, Brian; University of Oxford, Primary Care Health Sciences<br>Cartwright, Emma; Nanyang Technological University<br>Jebb, Susan; Univerof Oxford, Primary Care Health Sciences
**Primary Subject Heading**:	Public health
Secondary Subject Heading:	Nutrition and metabolism
Keywords:	PUBLIC HEALTH, NUTRITION & DIETETICS, Planetary Health, Meat consumption, Randomised Controlled Trial, Protocol

Replacing meat with alternative plant-based products (RE-MAPs): Protocol for a randomized controlled trial of a behavioural intervention to reduce meat consumption

Filippo Bianchi[1*], Paul Aveyard[1], Nerys Astbury[1], Brian Cook[1], Emma Cartwright[1], Susan A Jebb[1]

[1] Nuffield Department of Primary Care Health Science
* Corresponding Author: Filippo Bianchi, filippo.bianchi@phc.ox.ac.uk

Abstract

Introduction: Reducing meat consumption could contribute towards preventing some chronic conditions and protecting the natural environment. This study will examine the effectiveness of a behavioural intervention to reduce meat consumption.

Methods and analyses: Re-MAP is a randomised controlled trial comparing a behavioural intervention to reduce meat consumption with a no intervention control condition. Eligible volunteers will be recruited from the general public through advertisement and randomised in a 1:1 ratio to receive no intervention or a four-week intervention comprising the provision of free plant-based meat-alternatives, written information on the health and environmental benefits of eating less meat, success stories of people who reduced their meat consumption, and recipes. The primary outcome is the change in meat consumption at four weeks (T1) from baseline. Secondary and exploratory outcomes include changes in meat consumption at eight weeks (T2) from baseline and changes from the baseline to both follow-up in other aspects of participants diet, putative psychosocial determinants of eating a low meat diet and of using meat-substitutes, and biomarkers of health risk, including blood lipid profiles, blood pressure, weight, and body composition. Linear models will be employed to explore whether the changes in each of the aforementioned outcomes differ significantly between the control and intervention group. Qualitative interviews on a subsample of participants receiving the intervention will evaluate their experiences of the intervention and help to identify the mechanisms through which the intervention reduced meat consumption or the barriers preventing the intervention to aid this dietary transition.

Ethics and dissemination: The trial has been granted ethical approval by the Medical Sciences Interdivisional Research Ethics Committee (IDREC) of the University of Oxford (Ref: R54329/RE001). All results originating from this study will be submitted for publication in scientific journals and presented at meetings and through the media.

Trial registration number: ISRCTN13180635, Pre-recruitment.

For peer review only - http://bmjopen.bmj.com/site/about/guidelines.xhtml

Strengths and limitations of this study

Strengths

- The first randomised controlled trial assessing the behavioural, nutritional, psychosocial, and health impact of a four-week intervention to reduce meat consumption through replacement with meat-alternatives
- Assessment of putative psychosocial determinants of meat and meat-alternatives consumption will help to identify the active components of the intervention and will help inform future intervention development
- Health risk outcomes will provide preliminary evidence on potential health implications of replacing meat with meat-alternatives in the diet

Limitations

- Recruitment will occur among adult-only households within Oxford (UK), limiting the generalizability of the results
- The study will only provide proof of principle for the short-term effectiveness of a behavioural intervention to reduce meat consumption and future work will be needed to translate these insights into longer term-interventions in routine settings

For peer review only

For peer review only - http://bmjopen.bmj.com/site/about/guidelines.xhtml

Introduction

While meat is a source of important nutrients and can be part of a healthy diet (1), red and processed meat consumption is also associated with an increased risk of developing some forms of cancer (2–4), cardiovascular disease (5–8), and type-2-diabetes (7,9–11). Furthermore, producing meat can negatively affect the natural environment and contribute to anthropogenic global warming (12–14), which may also detrimentally affect human health (15–18). Reducing meat consumption could therefore help to promote public health and protect the natural environment, but a recent report identified "a remarkable lack of policies, initiatives or campaigns" designed to tackle the demand for meat (19). This state of inaction is partly due to the scarcity of evidence on the effectiveness of interventions to reduce meat consumption (19–23) warranting more experimental research to develop and evaluate such interventions. The rising availability of alternatives, such as textured vegetable proteins and mycoprotein-based alternatives (24) could help to reduce meat consumption, as these products resemble meat in their gastronomic function, appearance, and preparation. Nevertheless, uptake of meat-alternatives remains low in many developed countries (24–27), which might partly be due to a lack of familiarity with these foods (25,28,29). Interventions increasing people's familiarity with meat-alternatives could therefore help to overcome this barrier and, in turn, reduce meat consumption. A recent systematic review of experimental studies concluded that interventions that supplied plant-based alternatives were associated with reductions in meat consumption during, and several weeks after, the interventions (23). Nevertheless, this evidence is based on small uncontrolled pre-post intervention studies (30,31) and more systematic evaluations of the behavioural impact of such interventions is warranted. Additionally, there is currently no evidence from randomised trials on the psychosocial and health consequences of interventions aiming at reducing meat consumption through the replacement with meat-alternatives.

Objectives

The primary aim of the Replacing Meat with Alternative Plant-based products (RE-MAP) trial is to examine the effectiveness of a behavioural intervention to reduce meat consumption compared to a no intervention control condition. Additionally this study will evaluate the impact of the intervention on the consumption of other food groups, the nutritional composition of participants' diets, the putative psychosocial determinants of eating a low meat diet and of using plant-based meat-alternatives, and on biological markers of health risk, including blood lipid profiles, blood pressure, weight, and body composition. This study also aims to qualitatively investigate participants' experiences of the intervention, the mechanisms through which the intervention reduced meat consumption, and/or the barriers preventing the intervention to aid this dietary transition.

For peer review only - http://bmjopen.bmj.com/site/about/guidelines.xhtml

Methods

Study design and setting

The Re-MAP study will employ a two-arm parallel group individually randomised controlled trial to evaluate a four-week behavioural intervention to reduce meat consumption. The primary endpoint is defined as the change in average daily meat consumption at four weeks form baseline, assessed through self-reported seven days food diaries. The study will be conducted in Oxford, United Kingdom.

Recruitment

Participants will be recruited from the general population through advertisements in public buildings, newspapers, mailing lists, and social media. Individuals contacting the study team will receive a written information sheet summarizing the study protocol. Individuals confirming their interest will be called by the recruiting member of the research team, who will summarise the study protocol and answer any outstanding question. The recruiting member of the research team will also screen individuals against the eligibility criteria and invite eligible individuals to attend an enrolment appointment.

Eligibility criteria

Inclusion criteria:

(a) are ≥18 years old

(b) self-report to eat meat regularly

(c) belong to an adult-only household

(d) are willing to try meat-alternatives

(e) own adequate food storing facilities

(f) possess a device compatible with the requirements of the online food diary

(g) provide informed consent

Exclusion criteria:

(a) report they have relevant food allergies

(b) report suffering from an eating disorder

(c) report to be pregnant or plan pregnancy in the study period

(d) belong to the same household as a previously enrolled participant

(e) report consuming meat-alternatives more than once a week on average

(f) return baseline dietary records of insufficient quality for analysis

(g) the recruiting researcher deems the interested individual unable to adhere appropriately to the study protocol (e.g. insufficient knowledge of the English language, planned absences from main residence during the course of the study, enrolled in other longitudinal dietary intervention study).

For peer review only - http://bmjopen.bmj.com/site/about/guidelines.xhtml

Participant flow

Enrolment appointment

The enrolment appointment will take place on University premises. During this appointment an appropriately trained member of the research team will seek written informed consent (see supplementary file 1), witnessing this by means of dated signature. After gaining informed consent the enrolling member of the research team will set up participants' online food diaries to include six possible meal entries per day (breakfast, mid-morning, lunch, mid-afternoon, dinner, and post-dinner) and to allow the research team to remotely access participants' food diaries by means of a password. The recruiting member of the research team will also train participants in how to appropriately use the online food diaries and estimate portion sizes.

Baseline

Following the enrolment appointment, participants will complete a seven-day food diary over the week leading up to the following appointment, the baseline (T0). Participants not keeping sufficiently detailed diaries and those eating meat on less than five eating occasions over the week will be discontinued. At the baseline appointment an appropriately trained member of the research team will collect participants' food diaries, ask participants to answer the baseline online questionnaire, and measure participants blood lipids profile, blood pressure, weight, and body composition. At the end of the baseline appointment participants will be randomised to one of the two study conditions and will then follow the respective protocol for the next four weeks.

Follow up

Participants will be invited to attend a four-week (T1) and an eight-week (T2) follow-up and to keep a seven-days food diary over the week leading up to each follow-up. During the follow-up appointments a member of the research team will collect the respective food diary, ask participants to answer an online questionnaire, and measure participants blood lipids profile, blood pressure, weight, and body composition.

Sample size

Due to lack of research studies directly comparable to ours, pragmatic considerations have guided the decision to terminate recruitment once a sample of at least 100 volunteers have completed the four-weeks follow-up. A power analysis based on this pragmatically selected sample size suggests that 100 participants completing the primary outcome will allow detection of a medium effect size of d=0.6 with a power of 1-beta=0.84 and a two-tailed alpha criterion of 0.05.

Randomisation and blinding

Participants' group allocation will be based on a computer generated randomisation sequence, produced by an independent statistician. The randomisation sequence was designed to individually allocate participants to the intervention or control condition

For peer review only

in a 1:1 ratio and to achieve a proportional gender balance in the two conditions through blocking and stratification by sex. The research team is blinded to the randomisation sequence and to its block sizes and sequence. Allocation will be revealed to the researcher performing the randomisation only after the first food diary has been returned. Due to the nature of the intervention, participants and some members of the research team cannot be blind to participants' group allocation. The members of the research team analysing the food diaries will be blind to the group allocation. Due to the nature of the outcomes the risk of investigator bias will be low. To address the risk of social desirability bias in participants' reporting of foods intake and questionnaire responses, participants will be reminded during the enrolment visit and before each questionnaire that there are no right or wrong answers.

Intervention and comparator

Intervention

Re-MAP is a four-week behavioural intervention, which aims to reduce meat consumption, defined as non-seafood meat products, among regular meat eaters. Following an analysis of the target behaviour, i.e. a reduction in meat consumption, we included five psychosocial variables as the intervention's targets: attitudes, perceived behavioural control, and subjective social norms of eating a low meat diet, as well as attachment to meat, and eating identities (e.g. 'meat-eater' or 'vegetarian'). We then selected four intervention functions from the Behaviour Change Wheel (32,33) with the aim of influencing these psychosocial variables: (1) environmental restructuring enacted through providing meat-alternatives for four weeks, (2) training enacted through recipes, (3) education enacted through infographics on the health and environmental benefits of eating less meat, and (4) social modelling enacted through written vignettes outlining the story of people who reduced their meat consumption. These success stories were developed following an online patient and public involvement (PPI) activity. This PPI activity involved asking people who consciously reduced their consumption of meat to share their motives to do so, their strategies to enact this dietary transition, and the way they overcame the challenges associated with this transition. A logic model of the intervention is displayed in figure 1.

Insert here logic model

Figure 1: Intervention logic model

Comparator

Participants in the control condition will receive no intervention. The TiDIER checklist (34) for the Re-MAP intervention and the comparator is reported in table 1.

	Intervention	Comparator
BRIEF NAME	Re-MAP – a behavioural intervention to reduce meat consumption	No intervention
WHY	Environmental restructuring: Meat alternatives will be provided for one	N/A

For peer review only - http://bmjopen.bmj.com/site/about/guidelines.xhtml

	month with the aim of enhancing attitudes towards and behavioural control of eating a low meat diet by making meat-free alternative easily available to participants. This intervention component also aims to reduce participants' attachment to meat. Participants will select from a range of commercially available meat-alternatives including soy and other textured vegetable protein products (e.g. soy sausages), plant-based and pulses-based products (e.g. bean burgers), mycoprotein products (e.g. mycoprotein steaks). Meat alternatives will be defined as meat-free products that fulfil the same gastronomic function as products that normally contain meat (e.g. sausages, burgers, meatballs, steaks, or mince).<br><br>Training: Recipes will be provided with the aim of enhancing participants' behavioural control of eating a low meat diet by enhancing their skills of preparing meat-free meals.<br><br>Education: Information leaflets about the health and environmental benefits of eating less meat will be provided to enhance participants' attitudes towards eating a low meat diet and to reduce participants' attachment to meat.<br><br>Social modelling: written success stories of people who reduced their meat consumption will be provided to increase participants perceived social norm of eating a low meat diet and to promote the dietary identity of meat reducers, such as flexitarians.	
WHAT	Environmental restructuring: Participants will be provided with meat alternatives for one month, which they will be able to select from a printed catalogue of commercially available meat-alternatives. Participants will be asked to select enough meat-alternatives to have a meat-free product available on every occasion on which they would normally have meat for two weeks. Participants will be free to order enough foods to cater for themselves and other members of their household, if they wished to do so. The meat-alternatives will be delivered to participants' homes by a food retailer on up to two occasions over the intervention month: the first delivery will be scheduled immediately after participants are allocated to the intervention condition. The second delivery will be scheduled two weeks after the randomisation for participants who wish to top up their stock of meat-alternatives.<br><br>Training: A printed booklet containing 11 illustrated recipes of meat-alternatives will be delivered immediately after participants are allocated to the intervention condition. These recipes will incorporate some of the meat-alternatives used as part of this study. A second cookbook predominantly reporting on more general meat-free recipes (i.e. not focussing on meat alternatives) will be provided during the fourth intervention week. All participants received the same recipes.<br><br>Education: Participants will receive 8 printed pages of illustrated information on the health (4 pages) and environmental implications(4 pages) of eating less meat and 2 introduction pages and references delivered per post to their home over the course of the intervention month.. The info-graphics were developed using publicly available information from peer reviewed literature and relevant environmental or health organisations (e.g. cancer research UK). Immediately after being allocated to the intervention condition participants will receive an illustrated binder, which they will use to collect the information leaflets. The binder will include 2 pages of introductory information and the sources from which the information was drawn.<br><br>Success stories: Participants will receive three illustrated success stories vignettes delivered per post to their home during the last intervention week.	N/A

For peer review only

For peer review only - http://bmjopen.bmj.com/site/about/guidelines.xhtml

	The success stories will cover a range of different demographics (sex and age), different motives for eating less meat, and different strategies to transition to lower meat diets. The narratives will be about eating less meat rather than about ceasing to eat meat entirely. Participants will also receive a sheet on which they could report their own success story if the wish to do so. Participants will be asked to add this information to their illustrated binder.	
WHO	The lead researcher of this trial (FB) will deliver the intervention. An Access Database System will be used to schedule the deliveries of each intervention component ensuring that each intervention component will be delivered at the appropriate time for each participant.	N/A
HOW	The intervention consists in the delivery of the aforementioned materials. We will use the delivery services of one of UK's largest food retailers to purchase and deliver the meat-alternatives to participants. We will use Royal Mail to send printed materials. The binder will be delivered to participants immediately after they are randomised to the intervention condition.	N/A
WHERE	N/A	N/A
TAILORED	N/A	N/A
HOW Well	We elected to use a single study account with the food retailer to schedule all the study deliveries, which will enable us to monitor the successful completion and receipt of each delivery. Due to the nature of the intervention it will not be necessary to establish any other systems to monitor the fidelity of the intervention delivery.	N/A

Table 1: TiDIER checklist describing the Re-MAP intervention and no-intervention comparator

Patients and public involvement

Following the development of the basic intervention structure, we held a discussion group with ten members of the general public aiming to improve the acceptability and effectiveness of the RE-MAP intervention. We invited five meat eaters and five meat reducers to attend the discussion group, aiming to include people representing the target population of the intervention as well as people that successfully reduced their meat consumption. Public contributors were recruited using an established mailing list. The discussion group informed the development of each intervention component and of other aspects of the trial including:

- What type of meat-alternatives to offer as part of the intervention
- How to design the educational intervention components to be engaging and easily accessible to different publics
- What language to use as part of the success stories vignettes and how to increase their relatability
- What cookbooks and recipes to use as part of the intervention
- The likely burden of trial participation and how to best compensate trial participants

Contributors to the aforementioned public involvement activities will not be involved in other aspects of the trial implementation (such as recruitment) and will be asked not to enrol as trial participants, as they will have already reviewed much of the intervention material.

For peer review only

Outcomes

Primary outcome

- Change in mean daily grams of meat consumed between the baseline (T0) and the four-week follow-up (T1)

Secondary outcomes

- Change in mean daily grams of meat consumed between the baseline (T0) and the eight-week follow-up (T2)
- Change in the intention to eat a low meat diet between the baseline (T0) and both follow-up (T1, T2)
- Change in attachment to meat, eating identities, and in attitudes, perceived behavioural control, and subjective social norm of eating a low meat diet between the baseline (T0) and both follow-up (T1, T2)

Exploratory outcomes

- Change in participants' blood lipid profiles (total cholesterol, HDL cholesterol, triglycerides, LDL cholesterol, non-HDL cholesterol, LDL:HDL cholesterol ratio) between the baseline (T0) and both follow-up (T1, T2)
- Change in systolic and diastolic blood pressure between the baseline (T0) and both follow-up (T1, T2)
- Change in participants' body mass index between the baseline (T0) and both follow-up (T1, T2)
- Change in participants' body fat percentage between the baseline (T0) and both follow-up (T1, T2)
- Change in the number of meals containing foods from other food groups between the baseline (T0) and both follow-up (T1, T2)
- Change in participants' mean daily energy, macro-, and micronutrients intake between the baseline (T0) and both follow-up (T1, T2)
- Change in participants' intentions, attitudes, perceived behavioural control, and subjective social norms of using meat-alternatives between the baseline (T0) and both follow-up (T1, T2)
- Change in participants' desire for meat-substitutes to be similar to meat between the baseline (T0) and both follow-up (T1, T2)

Measurements

Table 2 provides a summary of the trial activities and of the measurement that will be collected at each stage of the trial.

	Visits				
	Telephone screening	Enrolment Visit	Baseline Visit	4 week follow up	8 week follow up
Enrolment					
Eligibility screening	X				
Informed consent		X			

For peer review only - http://bmjopen.bmj.com/site/about/guidelines.xhtml

Randomisation			X		
Intervention					
REMAP					
Control					
Demographic and psychosocial traits					
Demographics			X		
Food neophobia			X		
Self control scale			X		
Dietary measurements					
Food diary			X	X	X
Retrospective eating questionnaire			X	X	X
Psychosocial variables					
Attitude towards eating a low meat diet and using meat-alternatives			X	X	X
Perceived behavioural control of eating a low meat diet and using meat-alternatives			X	X	X
Subjective social norm of eating a low meat diet and using meat-alternatives			X	X	X
Intention to eat a low meat diet and to use meat-alternatives			X	X	X
Attachment to meat			X	X	X
Eating identity			X	X	X
Desire for similarity between meat and meat-alternatives			X	X	X
Biophysical outcomes					
Height			X		
Weight			X	X	X
Body composition			X	X	X
Blood pressure			X	X	X
Blood lipids profile			X	X	X
Qualitative work-stream					
Semi structured interviews					X

Table 2: Schedule of measurements and trial activities

Socio-demographic characteristics

- At the baseline, participants will be asked to self-report on their age, sex, highest degree, household income, household composition, ethnicity, and nationality

Psychological trait characteristics

- At the baseline, participants' trait food neophobia will be measured using a questionnaire scale adapted from Pliner and Hobden (35) including six items with a 7-point scale (disagree strongly – agree strongly)
- At the baseline, participants' self-control will also be assessed using a questionnaire scale adapted from Tangney et al. (36) including eight items with a 7-point scale (disagree strongly – agree strongly)

Dietary measurements

For peer review only - http://bmjopen.bmj.com/site/about/guidelines.xhtml

- Meat consumption will be measured in grams/day by disaggregating meat products recorded by participants on their seven-days food diaries. The daily average will exclude days in which energy intake was <1000kcal, which are considered unlikely to represent habitual consumption
- Average daily number of meals containing foods from other food groups will be measured counting the meals in participants' food diaries containing the food groups of interest, including:
    - Unprocessed pork meat
    - Unprocessed red meat from ruminants
    - Unprocessed poultry or game meat
    - Processed meat
    - Mycoprotein meat-alternatives
    - Soy-based meat-alternatives or meat-alternatives made of other textured vegetable protein
    - Other meat-alternatives (e.g. bean burgers)
    - Milk and yoghurt
    - Cheese
    - Dairy-free milk and yoghurt alternatives
    - Dairy-free cheese alternatives
    - Fish and seafood
    - Eggs
    - Pulses other than those in meat-alternatives
    - Vegetables other than those in meat-alternatives
    - Starchy foods other than those in meat-alternatives
    - Nuts and seeds other than those in meat-alternatives
    - Fruit
    - Savoury and sweet snacks
    - Soft drinks
    - Alcoholic drinks

  A retrospective eating questionnaire will also ask participants to recall the number of eating occasions on which they had the foods listed above over the week of their food diary. This questionnaire will only be used in sensitivity analyses
- The daily average energy intake and nutritional composition of participants' diets will be measured using data from the online food diary

Psychosocial variables

- Attachment to meat will be measured using the meat attachment questionnaire (37)
- Eating identities will be self-reported by participants among meat-eater, omnivore, flexitarian, pescatarian, vegetarian, vegan, or 'other'.
- Attitudes, subjective social norms, and perceived behavioural control to eat a low meat diet and to use meat-alternatives will be respectively assessed with

For peer review only - http://bmjopen.bmj.com/site/about/guidelines.xhtml

three questionnaire items constructed following Francis et al. (38) on a 7-point scale (disagree strongly – agree strongly)

- Intentions to eat a low meat diet and to use meat-alternatives will be assessed using a single questionnaire item on a 7-point scale (disagree strongly – agree strongly)
- Desire for similarity between meat and meat-alternatives will be assessed using 11 questionnaire items with a 7-point scale (disagree strongly – agree strongly) adapted from Hoek et al. (25)

Physical measures

- Blood lipids profiles (total cholesterol, HDL cholesterol, triglycerides, LDL cholesterol, non-HDL cholesterol, LDL:HDL cholesterol ratio) will be measured using Alere Cholestech LDX®
- Height will be measured to the nearest 0.1cm using a stadiometer
- Weight and body composition will be measured using an electronic scale (SC-240 MA, Tanita Japan), which records the proportion of body fat using bioelectrical impedance. Weight was recorded to the nearest 0.1kg
- Seated blood pressure will be measured as the average of the second and third reading of three seated readings

Retention

We will use reminder text messages to increase attendance to each of the four study appointments. Additionally participants will receive financial compensation for partaking in each of the 3 assessment visits. Participants will have the right to withdraw from the study at any time. The principal investigator will have the right to discontinue participants' involvement in the study when they become ineligible and/or when significant protocol deviations occur. The data of participants who withdraw will be kept and might be used in exploratory and sensitivity analyses, unless the participant requests for the data to be deleted.

Adverse events

Any study-related adverse event will be reported to the Research Ethics Committee in accordance to Good Clinical Practice (GCP). All study-related adverse events will be included in the final trial report.

Data management

Data will be entered by a trained member of the research team and stored in an OpenClinica database that was specifically developed for this trial. The database will feature ranges and validation checks to promote reliability in the data entry process. Data recording and storage will run in accordance with GCP.

Statistical analyses

We will employ linear models to investigate whether changes in meat consumption between the baseline and both follow-up differ significantly between the intervention

For peer review only - http://bmjopen.bmj.com/site/about/guidelines.xhtml

and the control group. Our main analysis will employ unadjusted models and only include data from participants completing the relevant follow-up. Sensitivity analysis will be performed with a Baseline Observation Carried Forward (BOCF) assumption for missing data and adjusting for baseline variables. The intervention effect will be reported with 95% CI and p-values. A two-tailed criterion p-value of alpha=0.05 will be used to assess the statistical significance of the results. The same procedure will be employed to assess whether changes in the other pre-specified dietary, nutritional, psychosocial, and biophysical outcomes between the baseline and both follow-up differ significantly between the control and the intervention group. Detailed main-, subgroup-, and sensitivity analyses plans will be finalised before conducting any specific outcome analysis. No interim analysis is planned.

Qualitative study

After the eight-week follow-up, a subsample of participants receiving the intervention will be invited to take part in a semi-structured interview. This qualitative study aims to understand participants' experiences of the intervention, the mechanisms through which the intervention helped reducing meat consumption, or the barriers preventing the intervention to aid this transition. The semi-structured interviews will follow a discussion guide while also remaining sensitive to unsolicited themes. The interview will set the context by asking participants to elaborate on their motivation to volunteer for the trial and on their thoughts and feelings towards reducing meat consumption prior to enrolling into the study. Participants will then be encouraged to elaborate on the mechanisms through which they felt that the intervention helped them eat less meat or the barriers preventing the intervention to do so. In doing so participants will be prompted to think about the intervention in its entirety as well as about each individual intervention component. Participants will be encouraged to elaborate on their perceived ability and motivation to maintain a lower consumption of meat after the intervention period and beyond the context of the study. Whenever possible we will use open questions to encourage participants to elaborate on their thoughts and feelings freely and in depth. We aim to avoid questions of evaluative nature to minimise the risk of social desirability bias. We anticipate interviewing 20 participants, however sampling will be extended should new themes emerge during the interviewing process. We will employ a purposeful sampling technique aiming to achieve a sex balance. Participants will be free to decide whether or not to be interviewed. No additional compensation will be offered to participants agreeing to be interviewed. Qualitative interviews will be conducted in person and transcribed verbatim. Transcriptions will be analysed using NVIVO and employing a data driven thematic analysis to identify codes and to group these codes into broader themes.

Trial steering committee

The principal investigator will be responsible for the project coordination and the senior investigators will oversee the operational aspects of the trial. The authors of this protocol will form the trial management group (TMG), which will regularly monitor the study implementation, as well as the data generation, documentation,

For peer review only - http://bmjopen.bmj.com/site/about/guidelines.xhtml

and reporting. All members of the TMG are trained in GCP and will take appropriate actions to safeguard participants and the quality of the trial. Access to data will be granted to appropriate members of the research team and to authorised representatives from the host institution to monitor and/or audit the study and ensure compliance with regulations.

Ethics and dissemination

The investigators will ensure that this study is conducted in accordance with the principles of the Declaration of Helsinki, with relevant institutional regulations, with GCP, and GDPR regulations. This study was reviewed and received ethical approval by the Medical Sciences Interdivisional Research Ethics Committee of the University of Oxford (R54329/RE001). Substantial planned changes to the protocol, an end of study notification, and a final report will be submitted to the aforementioned research ethics committee. The results of this RCT will be reported following the Consolidated Standards of Reporting Trials guidelines (39) and submitted for publication to scientific journals, regardless of the outcome. Authorship will be determined in accordance with the ICMJE guidelines. Contributors of other parties and funding will be acknowledged. Results will also be presented at national and international conferences and disseminated through established networks. A lay summary will be distributed through an established newsletter to which participants can subscribe on their last study appointment.

Sponsor

University of Oxford
University Offices
Wellington Square
Oxford
OX1 2JD
United Kingdom

The sponsor has no involvement in the implementation of the study.

Acknowledgements

We thank all the PPI contributors for having helped us develop the RE-MAP intervention. We thank Lynne Maddocks for her assistance in forming the PPI panel for this study. We thank Lucy Eldridge for her support in developing the study database. We thank Jason Oke for his assistance in developing the randomization sequence. We thank Alexa Hayley and Bernhard Haring for their comments on previous versions of this manuscript.

Authors' contributions: All authors have been involved in shaping each stage of this research protocol. FB has written this protocol and developed the intervention and led on the study design. FB, SAJ, and PA have designed the study. FB and NA have developed the trial management system. NA, BC, and EC have contributed in designing this research and the intervention.

For peer review only - http://bmjopen.bmj.com/site/about/guidelines.xhtml

Funding: This research is funded by the Wellcome Trust, Our Planet Our Health programme (Livestock, Environment and People - LEAP), award number 205212/Z/16/Z. FB's time on this project is funded by the Medical Research Council (MRC), Green Templeton College Oxford, and the National Institute for Health Research (NIHR) School for Primary Care Research (SPCR). EC's and BC's time on this project is funded by the Wellcome Trust, Our Planet Our Health programme (Livestock, Environment and People - LEAP), award number 205212/Z/16/Z. NA, PA, and SAJ are supported by the NIHR Oxford Biomedical Research Centre and Collaboration for Leadership in Applied Health Research and Care Oxford at Oxford Health NHS Foundation Trust. PA and SAJ are NIHR Senior Investigators.

Competing interests statement

The authors have no known competing interests to declare.

References

1. Scarborough P, Kaur A, Cobiac L, Owens P. Eatwell Guide: modelling the dietary and cost implications of incorporating new sugar and fibre guidelines. BMJ Open [Internet]. 2016 [cited 2017 May 31]; Available from: http://bmjopen.bmj.com/content/6/12/e013182.abstract
2. Bouvard V, Loomis D, Guyton KZ, Grosse Y, Ghissassi F El, Benbrahim-Tallaa L, et al. Carcinogenicity of consumption of red and processed meat. Lancet Oncol [Internet]. Elsevier; 2015 Dec 1 [cited 2017 Nov 9];16(16):1599–600. Available from: http://linkinghub.elsevier.com/retrieve/pii/S1470204515004441
3. Chan DSM, Lau R, Aune D, Vieira R, Greenwood DC, Kampman E, et al. Red and processed meat and colorectal cancer incidence: meta-analysis of prospective studies. PLoS One [Internet]. Public Library of Science; 2011 Jan 6 [cited 2015 Oct 26];6(6):e20456. Available from: http://journals.plos.org/plosone/article?id=10.1371/journal.pone.0020456
4. Parkin DM, Boyd L, Walker LC. 16. The fraction of cancer attributable to lifestyle and environmental factors in the UK in 2010. Br J Cancer [Internet]. 2011 Dec 6 [cited 2014 Oct 30];105 Suppl:S77-81. Available from: http://www.pubmedcentral.nih.gov/articlerender.fcgi?artid=3252065&tool=pmcentrez&rendertype=abstract
5. Schwingshackl L, Hoffmann G, Lampousi A-M, Knüppel S, Iqbal K, Schwedhelm C, et al. Food groups and risk of type 2 diabetes mellitus: a

For peer review only - http://bmjopen.bmj.com/site/about/guidelines.xhtml

systematic review and meta-analysis of prospective studies. Eur J Epidemiol [Internet]. Springer Netherlands; 2017 Apr 10 [cited 2017 May 23];1–13. Available from: http://link.springer.com/10.1007/s10654-017-0246-y

6. Micha R, Wallace SK, Mozaffarian D. Red and Processed Meat Consumption and Risk of Incident Coronary Heart Disease, Stroke, and Diabetes Mellitus: A Systematic Review and Meta-Analysis. Circulation [Internet]. 2010 Jun 1 [cited 2017 May 23];121(21):2271–83. Available from: http://www.ncbi.nlm.nih.gov/pubmed/20479151

7. Micha R, Michas G, Mozaffarian D. Unprocessed red and processed meats and risk of coronary artery disease and type 2 diabetes--an updated review of the evidence. Curr Atheroscler Rep [Internet]. 2012 Dec [cited 2015 Dec 27];14(6):515–24. Available from: http://www.pubmedcentral.nih.gov/articlerender.fcgi?artid=3483430&tool=pmcentrez&rendertype=abstract

8. Song M, Fung TT, Hu FB, Willett WC, Longo VD, Chan AT, et al. Association of Animal and Plant Protein Intake With All-Cause and Cause-Specific Mortality. JAMA Intern Med [Internet]. 2016 Oct 1 [cited 2018 Dec 6];176(10):1453. Available from: http://www.ncbi.nlm.nih.gov/pubmed/27479196

9. Feskens EJM, Sluik D, van Woudenbergh GJ. Meat Consumption, Diabetes, and Its Complications. Curr Diab Rep [Internet]. 2013 Apr 25 [cited 2017 Nov 9];13(2):298–306. Available from: http://www.ncbi.nlm.nih.gov/pubmed/23354681

10. Barnard N, Levin S, Trapp C. Meat consumption as a risk factor for type 2 diabetes. Nutrients [Internet]. Multidisciplinary Digital Publishing Institute (MDPI); 2014 Feb 21 [cited 2017 Nov 9];6(2):897–910. Available from: http://www.ncbi.nlm.nih.gov/pubmed/24566443

11. Pan A, Sun Q, Bernstein AM, Manson JE, Willett WC, Hu FB. Changes in Red Meat Consumption and Subsequent Risk of Type 2 Diabetes Mellitus. JAMA Intern Med [Internet]. 2013 Jul 22 [cited 2018 Dec 6];173(14):1328. Available from: http://www.ncbi.nlm.nih.gov/pubmed/23779232

12. Steinfeld H, Gerber P, Wassenaar T. Livestock's long shadow. Issues Options, … [Internet]. 2006 [cited 2017 May 23]; Available from: https://www.rfp-europe.org/fileadmin/SITE_ERFP/ERFP_meetings/2007_Dublin/Dublin2007_LivestocksLongShadow_HOFFMANN.pdf

For peer review only - http://bmjopen.bmj.com/site/about/guidelines.xhtml

13. Pimentel D, Pimentel M. Sustainability of meat-based and plant-based diets and the environment. Am J Clin Nutr [Internet]. American Society for Nutrition; 2003 Sep [cited 2017 May 23];78(3 Suppl):660S–663S. Available from: http://www.ncbi.nlm.nih.gov/pubmed/12936963

14. Tilman D, Clark M. Global diets link environmental sustainability and human health. Nature [Internet]. Nature Publishing Group, a division of Macmillan Publishers Limited. All Rights Reserved.; 2014 Nov 27 [cited 2015 Jul 27];515(7528):518–22. Available from: http://dx.doi.org/10.1038/nature13959

15. Watts N, Amann M, Ayeb-Karlsson S, Belesova K, Bouley T, Boykoff M, et al. The Lancet Countdown on health and climate change: from 25 years of inaction to a global transformation for public health. Lancet [Internet]. Elsevier; 2017 Oct 30 [cited 2018 Jan 25]; Available from: https://www.sciencedirect.com/science/article/pii/S0140673617324649?_rdoc=1&_fmt=high&_origin=gateway&_docanchor=&md5=b8429449ccfc9c30159a5f9aeaa92ffb

16. Mcmichael AJ, Campbell-Lendrum DH, Corvalán CF, Ebi KL, Githeko AK, Scheraga JD, et al. Climate change and human health. Risks and responses [Internet]. 2003 [cited 2018 Jan 25]. Available from: http://www.who.int/globalchange/publications/climchange.pdf

17. Economou V, Gousia P. Agriculture and food animals as a source of antimicrobial-resistant bacteria. Infect Drug Resist [Internet]. Dove Press; 2015 [cited 2018 Jan 25];8:49–61. Available from: http://www.ncbi.nlm.nih.gov/pubmed/25878509

18. Tang KL, Caffrey NP, Nóbrega DB, Cork SC, Ronksley PE, Barkema HW, et al. Restricting the use of antibiotics in food-producing animals and its associations with antibiotic resistance in food-producing animals and human beings: a systematic review and meta-analysis. Lancet Planet Heal [Internet]. Elsevier; 2017 Nov 1 [cited 2018 May 13];1(8):e316–27. Available from: http://www.ncbi.nlm.nih.gov/pubmed/29387833

19. Bailey R, Froggatt A WL. Livestock–climate change's forgotten sector. [Internet]. 2014 [cited 2017 Nov 10]. Available from: https://scholar.google.co.uk/scholar?hl=en&as_sdt=0%2C5&q=Livestock+–+Climate+Change's+Forgotten+Sector+Global+Public+Opinion+on+Meat

For peer review only - http://bmjopen.bmj.com/site/about/guidelines.xhtml

+and+Dairy+Consumption&btnG=

20. Garnett T, Mathewson S, Angelides P, Borthwick F. Policies and actions to shift eating patterns: What works? [Internet]. 2015 [cited 2017 May 24]. Available from: http://www.fcrn.org.uk/sites/default/files/fcrn_chatham_house_0.pdf

21. Wellesley L, Happer C, Froggatt A. Chatham House Report Changing Climate, Changing Diets Pathways to Lower Meat Consumption. 2015 [cited 2017 Nov 10]; Available from: https://www.chathamhouse.org/sites/files/chathamhouse/publications/research/CHHJ3820 Diet and climate change 18.11.15_WEB_NEW.pdf

22. Bianchi F, Dorsel C, Garnett E, Aveyard P, Jebb SA. Interventions targeting conscious determinants of human behaviour to reduce the demand for meat: a systematic review with qualitative comparative analysis. Int J Behav Nutr Phys Act [Internet]. BioMed Central; 2018 Dec 19 [cited 2018 Dec 6];15(1):102. Available from: https://ijbnpa.biomedcentral.com/articles/10.1186/s12966-018-0729-6

23. Bianchi F, Garnett E, Dorsel C, Aveyard P, Jebb SA. Restructuring physical micro-environments to reduce the demand for meat: a systematic review and qualitative comparative analysis. Lancet Planet Heal [Internet]. Elsevier; 2018 Sep 1 [cited 2018 Dec 6];2(9):e384–97. Available from: http://www.ncbi.nlm.nih.gov/pubmed/30177007

24. Mintel Group Ltd. Meat-free Foods - UK [Internet]. 2017 [cited 2017 Nov 12]. Available from: http://academic.mintel.com/sinatra/oxygen_academic/list/id=796253&type=RCItem#0_1___page_RCItem=0

25. Hoek A, Luning P, Weijzen P, Engels W, Kok F. Replacement of meat by meat substitutes. A survey on person-and product-related factors in consumer acceptance. Appetite [Internet]. 2011 [cited 2017 May 24]; Available from: http://www.sciencedirect.com/science/article/pii/S0195666311000523

26. Hartmann C, Siegrist M. Consumer perception and behaviour regarding sustainable protein consumption: A systematic review. Trends Food Sci Technol [Internet]. 2017 Mar [cited 2017 Nov 26];61(61):11–25. Available from: http://linkinghub.elsevier.com/retrieve/pii/S0924224416302904

27. Kumar P, Chatli MK, Mehta N, Singh P, Malav OP, Verma AK. Critical Reviews in Food Science and Nutrition Meat analogues: Health promising

For peer review only - http://bmjopen.bmj.com/site/about/guidelines.xhtml

sustainable meat substitutes Meat analogues: Health promising sustainable meat substitutes. 2017 [cited 2018 May 16]; Available from: http://www.tandfonline.com/action/journalInformation?journalCode=bfsn20

28. Clery E, Bailey R. Food technologies Findings from the 2008 British Social Attitudes survey. Food Technol Find from 2008 Br [Internet]. 2010 [cited 2017 May 24]; Available from: http://www.fcrn.org.uk/sites/default/files/BSA_Food_technologies_findings.pdf

29. Hoek AC, Elzerman JE, Hageman R, Kok FJ, Luning PA, Graaf C de. Are meat substitutes liked better over time? A repeated in-home use test with meat substitutes or meat in meals. Food Qual Prefer [Internet]. Elsevier; 2013 Apr 1 [cited 2018 May 13];28(1):253–63. Available from: https://www.sciencedirect.com/science/article/abs/pii/S0950329312001280

30. Clark M. Chronic Effects Of Replacing Red And Processed Meat With Non/Reduced Meat Alternatives [Internet]. Cambridge University Press; 2017 [cited 2017 Nov 12]. Available from: https://www.cambridge.org/core/product/identifier/S0029665117001434/type/journal_article

31. Holloway T, Salter AM, McCullough FS. Dietary intervention to reduce meat intake by 50% in University students – a pilot study. Proc Nutr Soc [Internet]. Cambridge University Press; 2012 Jan 19 [cited 2017 Nov 12];71(OCE2):E164. Available from: http://www.journals.cambridge.org/abstract_S0029665112002212

32. Michie S, Atkins L, West R. The behaviour change wheel: a guide to designing interventions. Needed: physician leaders [Internet]. 2014 [cited 2017 May 24]; Available from: http://www.physicianleaders.ca/assets/cspljournalsummer2015.pdf#page=26

33. Michie S, Stralen M van. The behaviour change wheel: a new method for characterising and designing behaviour change interventions. 2011 [cited 2017 May 24]; Available from: https://implementationscience.biomedcentral.com/articles/10.1186/1748-5908-6-42

34. Hoffmann TC, Glasziou PP, Boutron I, Milne R, Perera R, Moher D, et al.

For peer review only - http://bmjopen.bmj.com/site/about/guidelines.xhtml

Better reporting of interventions: template for intervention description and replication (TIDieR) checklist and guide. BMJ [Internet]. 2014 [cited 2017 Sep 1];348. Available from: http://www.bmj.com/content/348/bmj.g1687

35. Pliner P, Hobden K. Development of a scale to measure the trait of food neophobia in humans. Appetite [Internet]. 1992 Oct [cited 2018 May 13];19(2):105–20. Available from: http://www.ncbi.nlm.nih.gov/pubmed/1489209

36. Tangney J, … AB-S-R and, 2018 undefined. High self-control predicts good adjustment, less pathology, better grades, and interpersonal success. taylorfrancis.com [Internet]. [cited 2018 May 13]; Available from: https://www.taylorfrancis.com/books/e/9781351707756/chapters/10.4324%2F9781315175775-12

37. Graça J, Calheiros MM, Oliveira A. Attached to meat? (Un)Willingness and intentions to adopt a more plant-based diet. Appetite [Internet]. 2015 Dec [cited 2017 Nov 13];95:113–25. Available from: http://www.ncbi.nlm.nih.gov/pubmed/26148456

38. Francis J, Eccles M, Johnston M, Walker A. Constructing questionnaires based on the theory of planned behaviour: A manual for health services researchers. 2004 [cited 2018 May 13]; Available from: http://openaccess.city.ac.uk/1735/1/TPB Manual FINAL May2004.pdf

39. Schulz KF, Altman DG, Moher D. CONSORT 2010 Statement: updated guidelines for reporting parallel group randomised trials. BMC Med [Internet]. 2010 Jan [cited 2015 Mar 14];8(1):18. Available from: http://www.biomedcentral.com/1741-7015/8/18

For peer review only

For peer review only - http://bmjopen.bmj.com/site/about/guidelines.xhtml

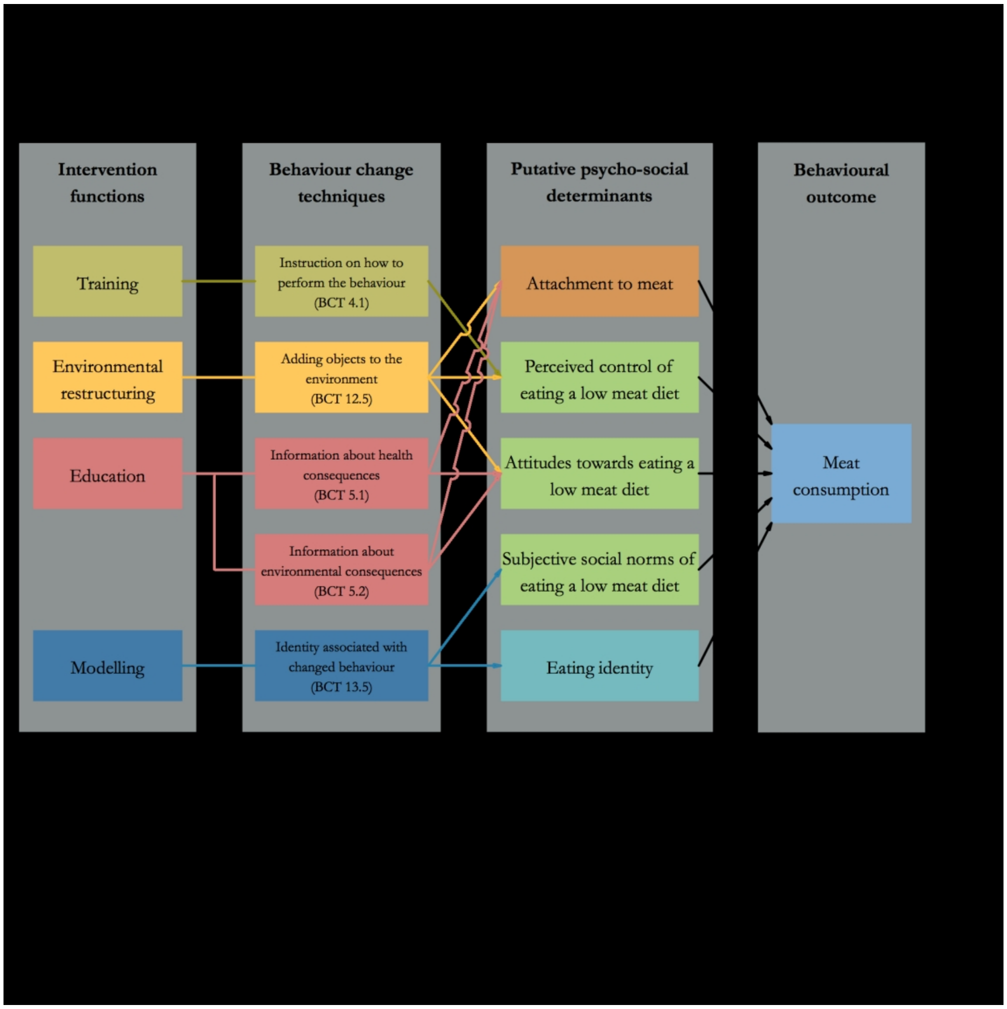
Intervention functions
Behaviour change techniques
Putative psycho-social determinants
Behavioural outcome
Training
Instruction on how to perform the behaviour (BCT 4.1)
Attachment to meat
Environmental restructuring
Adding objects to the environment (BCT 12.5)
Perceived control of eating a low meat diet
Education
Information about health consequences (BCT 5.1)
Attitudes towards eating a low meat diet
Meat consumption
Information about environmental consequences (BCT 5.2)
Subjective social norms of eating a low meat diet
Modelling
Identity associated with changed behaviour (BCT 13.5)
Eating identity

NUFFIELD DEPARTMENT OF PRIMARY CARE HEALTH SCIENCES

Principal Researcher: Filippo Bianchi
Contact Details: RE-MAP@phc.ox.ac.uk
Departmental web page: www.phc.ox.ac.uk
Department's address: Nuffield Department of Primary Care health Sciences, Radcliffe Observatory Quarter, Woodstock Road, Oxford. OX2 6GG
University web page: www.ox.ac.uk

Participant ID: ___________________________________

Replacing **M**eat with **A**lternative **P**rotein **S**ources (RE-MAP STUDY)

Participant Consent Form. CUREC-Approval Reference: R54329/RE001

The purpose of this study is to test the effectiveness of a behavioural intervention designed to help people reduce their consumption of meat.

Please initial the boxes to confirm you agree

I confirm that I have read and understood the information sheet for the above study. I have had the opportunity to consider the information, ask questions and have had these answered satisfactorily.	☐
I understand that my participation is voluntary and that I am free to withdraw at any time, without having to give any reason, and without any adverse consequences.	☐
I understand that designated individuals may look at research data collected during the study where it is relevant to my taking part in this study. I give permission for these individuals to access my data.	☐
I understand that this project has been reviewed by, and received ethics clearance through, the University of Oxford Central University Research Ethics Committee.	☐
I understand who will have access to personal data provided, how the data will be stored, and what will happen to the data at the end of the project.	☐
I understand that three finger prick blood samples will be taken from me during this study to measure blood cholesterol. I understand the procedure that will be used to carry out these analyses.	☐

For peer review only - http://bmjopen.bmj.com/site/about/guidelines.xhtml

I understand this research will be written up as a student's thesis, I understand how personal data included in that thesis will be published and stored.	☐
I understand that I may be quoted in an anonymous way in publications pertinent to this study and that I will *not* be identified personally in any of these publications.	☐
I understand how to raise a concern or make a complaint.	☐
I consent to being audio recorded.	☐
I understand how audio recordings will be used in research outputs	☐
I consent to take part in the above study.	☐
Optional: Should I be allocated to the intervention group, I understand and consent for my name, address, telephone number, and selection of meat substitutes to be shared with Sainsbury's to carry out the food deliveries.	☐
Optional: I agree for research data collected in this study to be given to researchers, including those working outside of the EU, to be used in other research studies. I understand that any data that leave the research group will be fully anonymised so that I cannot be identified.	☐

Name of participant　　Date　　Signature

Name of person taking consent　　Date　　Signature

For peer review only - http://bmjopen.bmj.com/site/about/guidelines.xhtml

STANDARD PROTOCOL ITEMS: RECOMMENDATIONS FOR INTERVENTIONAL TRIALS

SPIRIT 2013 Checklist: Recommended items to address in a clinical trial protocol and related documents*

Section/item	Item No	Description
**Administrative information**		
Title	1	Descriptive title identifying the study design, population, interventions, and, if applicable, trial acronym<br>P.1
Trial registration	2a	Trial identifier and registry name. If not yet registered, name of intended registry<br>P.1
	2b	All items from the World Health Organization Trial Registration Data Set<br>Provided in the protocol and in the ISRCTN trial registration (http://www.isrctn.com/ISRCTN13180635?q=filippo%20bianchi&filters=&sort=&offset=1&totalResults=1&page=1&pageSize=10&searchType=basic-search)
Protocol version	3	Date and version identifier<br>**Date applied**<br>22/06/2018<br>**Registered Online**<br>25/06/2018<br>**Published**<br>tbc
Funding	4	Sources and types of financial, material, and other support<br>P.14
Roles and responsibilities	5a	Names, affiliations, and roles of protocol contributors<br>P.1, P.14
	5b	Name and contact information for the trial sponsor<br>P.14
	5c	Role of study sponsor and funders, if any, in study design; collection, management, analysis, and interpretation of data; writing of the report; and the decision to submit the report for publication, including whether they will have ultimate authority over any of these activities<br>P.14

For peer review only - http://bmjopen.bmj.com/site/about/guidelines.xhtml

	5d	Composition, roles, and responsibilities of the coordinating centre, steering committee, endpoint adjudication committee, data management team, and other individuals or groups overseeing the trial, if applicable (see Item 21a for data monitoring committee) P.13
**Introduction**		
Background and rationale	6a	Description of research question and justification for undertaking the trial, including summary of relevant studies (published and unpublished) examining benefits and harms for each intervention P.3
	6b	Explanation for choice of comparators P.3
Objectives	7	Specific objectives or hypotheses P.3
Trial design	8	Description of trial design including type of trial (eg, parallel group, crossover, factorial, single group), allocation ratio, and framework (eg, superiority, equivalence, noninferiority, exploratory) P.4
**Methods: Participants, interventions, and outcomes**		
Study setting	9	Description of study settings (eg, community clinic, academic hospital) and list of countries where data will be collected. Reference to where list of study sites can be obtained P.4
Eligibility criteria	10	Inclusion and exclusion criteria for participants. If applicable, eligibility criteria for study centres and individuals who will perform the interventions (eg, surgeons, psychotherapists) P.4
Interventions	11a	Interventions for each group with sufficient detail to allow replication, including how and when they will be administered P. 6-8
	11b	Criteria for discontinuing or modifying allocated interventions for a given trial participant (eg, drug dose change in response to harms, participant request, or improving/worsening disease) N/A

For peer review only

	11c	Strategies to improve adherence to intervention protocols, and any procedures for monitoring adherence (eg, drug tablet return, laboratory tests)<br>P.12
	11d	Relevant concomitant care and interventions that are permitted or prohibited during the trial<br>P.4
Outcomes	12	Primary, secondary, and other outcomes, including the specific measurement variable (eg, systolic blood pressure), analysis metric (eg, change from baseline, final value, time to event), method of aggregation (eg, median, proportion), and time point for each outcome. Explanation of the clinical relevance of chosen efficacy and harm outcomes is strongly recommended<br>P. 8-12
Participant timeline	13	Time schedule of enrolment, interventions (including any run-ins and washouts), assessments, and visits for participants. A schematic diagram is highly recommended (see Figure)<br>P. 9-10
Sample size	14	Estimated number of participants needed to achieve study objectives and how it was determined, including clinical and statistical assumptions supporting any sample size calculations<br>P. 5
Recruitment	15	Strategies for achieving adequate participant enrolment to reach target sample size<br>P.4

Methods: Assignment of interventions (for controlled trials)

Allocation:

Sequence generation	16a	Method of generating the allocation sequence (eg, computer-generated random numbers), and list of any factors for stratification. To reduce predictability of a random sequence, details of any planned restriction (eg, blocking) should be provided in a separate document that is unavailable to those who enrol participants or assign interventions<br>P. 5-6
Allocation concealment mechanism	16b	Mechanism of implementing the allocation sequence (eg, central telephone; sequentially numbered, opaque, sealed envelopes), describing any steps to conceal the sequence until interventions are assigned<br>P. 5-6

For peer review only - http://bmjopen.bmj.com/site/about/guidelines.xhtml

Implementation	16c	Who will generate the allocation sequence, who will enrol participants, and who will assign participants to interventions<br>P. 5-6
Blinding (masking)	17a	Who will be blinded after assignment to interventions (eg, trial participants, care providers, outcome assessors, data analysts), and how<br>P. 5-6
	17b	If blinded, circumstances under which unblinding is permissible, and procedure for revealing a participant's allocated intervention during the trial<br>P. 5-6

Methods: Data collection, management, and analysis

Data collection methods	18a	Plans for assessment and collection of outcome, baseline, and other trial data, including any related processes to promote data quality (eg, duplicate measurements, training of assessors) and a description of study instruments (eg, questionnaires, laboratory tests) along with their reliability and validity, if known. Reference to where data collection forms can be found, if not in the protocol<br>P. 5, P. 9-12
	18b	Plans to promote participant retention and complete follow-up, including list of any outcome data to be collected for participants who discontinue or deviate from intervention protocols<br>P. 12
Data management	19	Plans for data entry, coding, security, and storage, including any related processes to promote data quality (eg, double data entry; range checks for data values). Reference to where details of data management procedures can be found, if not in the protocol<br>P. 12
Statistical methods	20a	Statistical methods for analysing primary and secondary outcomes. Reference to where other details of the statistical analysis plan can be found, if not in the protocol<br>P. 12
	20b	Methods for any additional analyses (eg, subgroup and adjusted analyses)<br>P. 12
	20c	Definition of analysis population relating to protocol non-adherence (eg, as randomised analysis), and any statistical methods to handle missing data (eg, multiple imputation)<br>P. 12

Methods: Monitoring

Data monitoring	21a	Composition of data monitoring committee (DMC); summary of its role and reporting structure; statement of whether it is independent from the sponsor and competing interests; and reference to where further details about its charter can be found, if not in the protocol. Alternatively, an explanation of why a DMC is not needed<br>P.13
	21b	Description of any interim analyses and stopping guidelines, including who will have access to these interim results and make the final decision to terminate the trial<br>P.12
Harms	22	Plans for collecting, assessing, reporting, and managing solicited and spontaneously reported adverse events and other unintended effects of trial interventions or trial conduct<br>P.12
Auditing	23	Frequency and procedures for auditing trial conduct, if any, and whether the process will be independent from investigators and the sponsor<br>P.13
**Ethics and dissemination**		
Research ethics approval	24	Plans for seeking research ethics committee/institutional review board (REC/IRB) approval<br>P.13
Protocol amendments	25	Plans for communicating important protocol modifications (eg, changes to eligibility criteria, outcomes, analyses) to relevant parties (eg, investigators, REC/IRBs, trial participants, trial registries, journals, regulators)<br>P.14
Consent or assent	26a	Who will obtain informed consent or assent from potential trial participants or authorised surrogates, and how (see Item 32)<br>P. 5
	26b	Additional consent provisions for collection and use of participant data and biological specimens in ancillary studies, if applicable<br>N/A
Confidentiality	27	How personal information about potential and enrolled participants will be collected, shared, and maintained in order to protect confidentiality before, during, and after the trial<br>P.12, 13
Declaration of interests	28	Financial and other competing interests for principal investigators for the overall trial and each study site<br>P. 15

For peer review only - http://bmjopen.bmj.com/site/about/guidelines.xhtml

Access to data	29	Statement of who will have access to the final trial dataset, and disclosure of contractual agreements that limit such access for investigators<br>P. 13
Ancillary and post-trial care	30	Provisions, if any, for ancillary and post-trial care, and for compensation to those who suffer harm from trial participation<br>N/A
Dissemination policy	31a	Plans for investigators and sponsor to communicate trial results to participants, healthcare professionals, the public, and other relevant groups (eg, via publication, reporting in results databases, or other data sharing arrangements), including any publication restrictions<br>P. 14
	31b	Authorship eligibility guidelines and any intended use of professional writers<br>P. 13-14
	31c	Plans, if any, for granting public access to the full protocol, participant-level dataset, and statistical code<br>Data will be provided on reasonable requests.
**Appendices**		
Informed consent materials	32	Model consent form and other related documentation given to participants and authorised surrogates<br>Attached
Biological specimens	33	Plans for collection, laboratory evaluation, and storage of biological specimens for genetic or molecular analysis in the current trial and for future use in ancillary studies, if applicable<br>N/A

*It is strongly recommended that this checklist be read in conjunction with the SPIRIT 2013 Explanation & Elaboration for important clarification on the items. Amendments to the protocol should be tracked and dated. The SPIRIT checklist is copyrighted by the SPIRIT Group under the Creative Commons "Attribution-NonCommercial-NoDerivs 3.0 Unported" license.

For peer review only
